# Advanced Nanomaterial Platforms for Targeted Therapy of Myocardial Ischemia–Reperfusion Injury

**DOI:** 10.34133/research.0822

**Published:** 2025-08-05

**Authors:** Hanxi Wang, Li Li, Lihan Luo, Yuqi Cheng, Feng Zhou, ShuangJun Zhao, Yang Li, Yuzhu Yang, Qianqian Zhou, Hanyun Niu, Jiannan He, Cao Zhang, Jian Guo, Longguang Tang, Jianhong Xu

**Affiliations:** ^1^ Department of Anesthesiology, the Fourth Affiliated Hospital of School of Medicine, and International School of Medicine, International Institutes of Medicine, Zhejiang University, Yiwu 322000, China.; ^2^ Center for Regeneration and Aging Medicine, the Fourth Affiliated Hospital of School of Medicine, and International School of Medicine, International Institutes of Medicine, Zhejiang University, Yiwu 322000, China.

## Abstract

Ischemic heart disease (IHD) remains a major global health challenge due to its persistently high incidence and mortality rates. Although early thrombolytic or interventional therapy reduces infarct size, myocardial ischemia–reperfusion injury (MIRI) often occurs when restoring blood flow to ischemic myocardium, paradoxically causing cardiomyocyte death. Unlike conventional cardioprotective agents, bioengineered nanomaterials enable targeted drug delivery to ischemic cardiomyocytes through tunable physicochemical properties. This improves therapeutic efficacy while reducing systemic exposure, providing innovative strategies for MIRI treatment. This review summarizes recent advances in nanomaterial-based MIRI therapies and critically evaluates their clinical translation potential, highlighting both opportunities and challenges.

## Introduction

Ischemic heart disease (IHD) remains the leading contributor to global cardiovascular mortality, with an estimated 9 million fatalities annually [1]. The global burden of IHD encompasses 200 million prevalent cases, demonstrating a case fatality rate of 4.5% [2]. Myocardial hypoxia due to coronary occlusion induces irreversible cardiomyocyte necrosis, progressing to myocardial infarction (MI) [3]. Current revascularization strategies, including thrombolysis and percutaneous coronary intervention (PCI), enable timely myocardial reperfusion; however, this therapeutic approach only partially mitigates infarct expansion [4]. This reperfusion process paradoxically induces pathological oxidative cascades and programmed cell death, clinically termed myocardial ischemia–reperfusion injury (MIRI) [5]. Conventional pharmacotherapy faces dual delivery challenges: nonspecific systemic biodistribution and cardiac-specific anatomical barriers, resulting in subtherapeutic drug accumulation (<5% injected dose/g) in ischemic myocardium [6]. Dose escalation strategies (e.g., >5 mg/kg) may improve efficacy but concomitantly elevate systemic toxicity risks. These limitations underscore the critical demand for targeted delivery systems, where nanomedicine provides a revolutionary solution to this delivery dilemma.

**Fig. 1.**
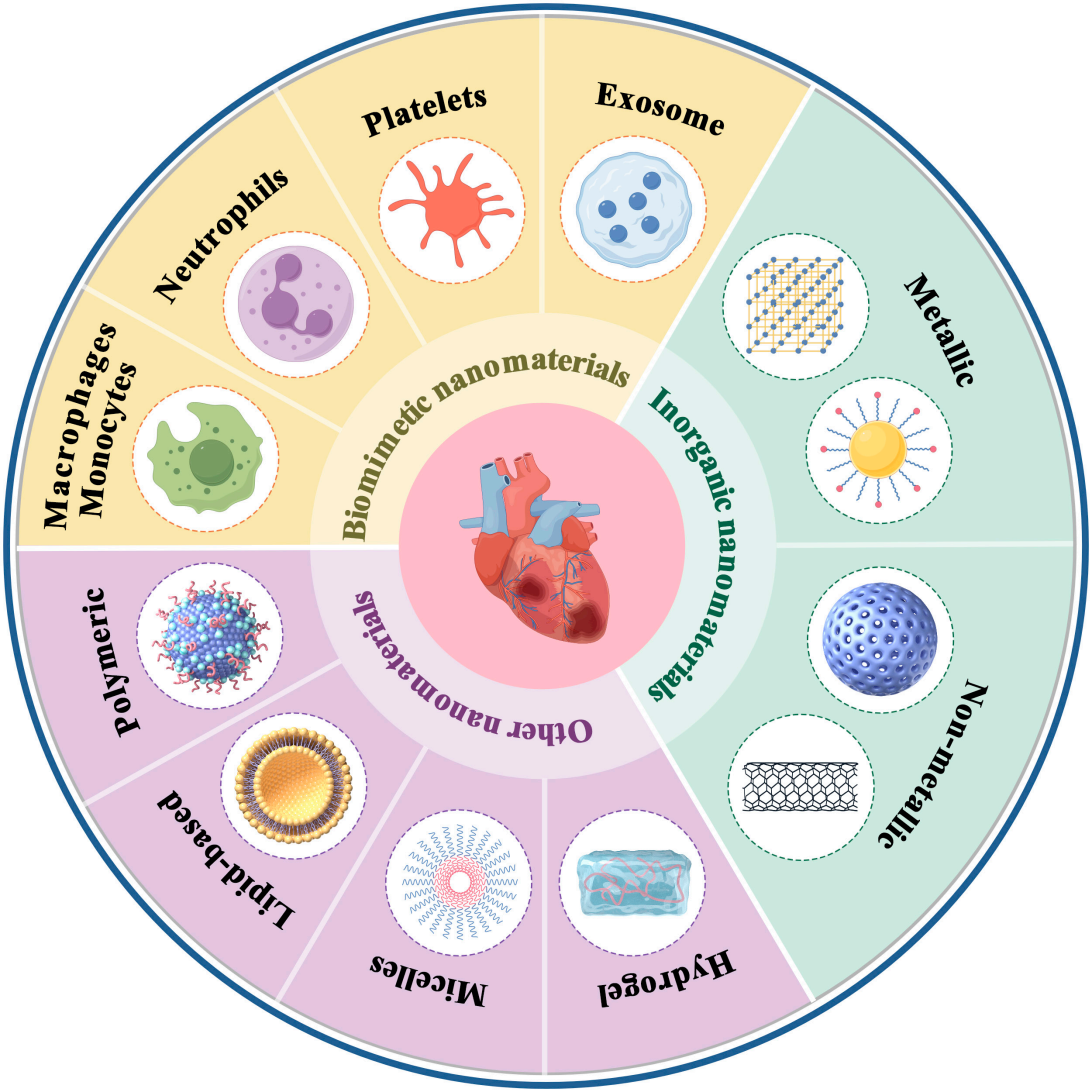
Schematic of various nanomaterials for MIRI therapy. (By Figdraw.)

Nanomedicine, defined as nanotechnology applications for biological system diagnosis and treatment, has witnessed remarkable research advancements in recent years. Nanomaterials, with sizes ranging from 1 to 100 nm, demonstrate distinctive physicochemical properties including structural versatility and multifunctional imaging capabilities [7]. These materials have been applied in many medical fields, such as targeted delivery of therapeutic or imaging agents across biological barriers to specific diseased tissues, high-precision disease detection through advanced imaging modalities, and molecular profiling via sensitive high-throughput methods [8]. The efficacy of nanomaterial platforms hinges on key structural parameters—size, surface charge, morphology, and functionalization strategy. These collectively govern biodistribution patterns, cellular uptake efficiency, and spatiotemporal drug release kinetics, critically determining targeting precision and cardioprotective outcomes in MIRI therapy. Through precise parameter engineering, researchers can tailor nanoplatforms for specific therapeutic objectives [9]. For instance, surface functionalization with targeting ligands (e.g., peptides and antibodies) enhances tissue-specific delivery and intralesional drug accumulation while minimizing off-target effects and systemic toxicity [10]. Nanocarrier-mediated drug delivery offers distinct advantages over free drugs: enhanced cellular permeability, protection from enzymatic degradation, optimized pharmacokinetic profiles, and prolonged systemic circulation [11]. Current MIRI therapeutic platforms encompass diverse nanomaterials ranging from lipid-based systems to metallic nanoparticles and biomimetic scaffolds. Timely synthesis of these advancements is crucial for guiding future research. This comprehensive review analyzes cutting-edge developments in nanomaterial-based MIRI interventions through 3 focused dimensions. First, we outline the key pathophysiological mechanisms underlying MIRI. Next, we critically evaluate the unique advantages of different nanomaterial classes and their emerging applications in MIRI management (Fig. 1). Finally, we discuss current translational challenges and propose clinically oriented development strategies to bridge the gap between bench and bedside.

## Pathophysiological Mechanism of MIRI

MIRI involves interconnected pathological cascades, with core mechanisms including inflammatory activation, oxidative stress, calcium dyshomeostasis, ferroptosis, mitochondrial dysfunction, and microvascular obstruction. Emerging evidence reveals that these pathways form a self-amplifying network driving cardiomyocyte death via epigenetic reprogramming, metabolic dysregulation, and dysregulated immune crosstalk. Contemporary nanotherapeutic strategies precisely target these specific pathophysiological processes utilizing engineered materials, with focused attention on novel therapeutic targets.

### Inflammation

MI healing progresses through 3 overlapping phases: inflammatory response, proliferative remodeling, and tissue maturation [12]. The inflammatory phase serves as the pivotal initiator of pathological progression through dual-activation mechanisms. Ischemia–reperfusion triggers sterile necrosis, initiating pattern recognition receptor-mediated immune activation. Necrotic cardiomyocytes release damage-associated molecular patterns (DAMPs), notably high-mobility group box 1 (HMGB1), S100 proteins, fibronectin, heat shock proteins, and mitochondrial DNA [13–15]. These DAMPs engage cardiac-expressed pattern recognition receptors, including Toll-like receptors (TLRs), NOD-like receptors (NLRs), receptor for advanced glycation end-products (RAGE), and complement receptors, orchestrating myeloid cell recruitment from hematopoietic reservoirs to infarct zones. This interaction activates downstream nuclear factor-κB (NF-κB) and mitogen-activated protein kinase signaling cascades, up-regulating pro-inflammatory mediators including tumor necrosis factor-α (TNF-α), interleukin-1β (IL-1β), IL-6, and IL-18 that establish autocrine amplification loops. This robust inflammatory response both initiates and amplifies core pathological mechanisms in MIRI. Infiltrating neutrophils and activated macrophages generate abundant reactive oxygen species (ROS), exacerbating oxidative stress. This process further activates inflammatory pathways and induces mitochondrial dysfunction. Crucially, neutrophils form endothelial-adherent clusters through CD11b/CD18 (Mac-1) integrin binding, worsening microvascular obstruction via mechanical occlusion and ROS release. Moreover, the inflammatory milieu promotes pathological processes such as intracellular calcium dyshomeostasis and ferroptosis. Emerging evidence indicates that amino acid metabolism orchestrates immune cell polarization and cytokine production in ischemic myocardium [16]. Targeting metabolic checkpoints (e.g., arginine and tryptophan pathways) offers novel avenues for inflammation resolution.

### Oxidative stress

Physiological levels of ROS serve as critical secondary messengers regulating cellular differentiation, proliferation, apoptosis, and homeostatic maintenance. Pathological ROS overload induces oxidative damage through lipid peroxidation, protein carbonylation, and DNA strand breaks in cardiomyocytes. Major ROS subtypes (O_2_·^−^, H_2_O_2_, ·OH) originate from mitochondrial electron transport chains, nicotinamide adenine dinucleotide phosphate oxidase, cytochrome P-450 systems, and peroxisomal oxidases [17]. ROS primarily contributes to MIRI via 3 key mechanisms. Firstly, direct oxidation of cellular components induces membrane integrity loss and organelle dysfunction—particularly mitochondrial damage—which triggers programmed necrosis and apoptosis. Such mitochondrial damage amplifies ROS generation, establishing a self-perpetuating oxidative stress cycle. Secondly, ROS activates redox-sensitive transcription factors that up-regulate TNF-α, IL-1β, and IL-6 [18]. These cytokines synergistically activate the NF-κB pathway, initiating pro-death transcriptional programs [19]. In addition, the excessive production of ROS at the site of injury can also cascade with nitric oxide (NO) to generate cytotoxic peroxynitrite anion (ONOO^−^), inducing tyrosine nitration and mitochondrial permeability transition pore (mPTP) opening. Critically, ONOO^−^ itself is a potent oxidant that directly damages cellular components and further impairs mitochondrial function, amplifying the initial injury. Sun et al. [20] engineered self-sustaining selenium-embedded nanoparticles (SSSe NPs) to target the ROS–inflammation–ferroptosis cascade. These nanoparticles passively target damaged mitochondria and scavenge multiple ROS subtypes—including O₂·^−^, H₂O₂, and ·OH—restoring glutathione peroxidase 4 (GPX4) activity. By disrupting this pathological cascade, they confer comprehensive protection against MIRI.

### Calcium overload

**Fig. 2.**
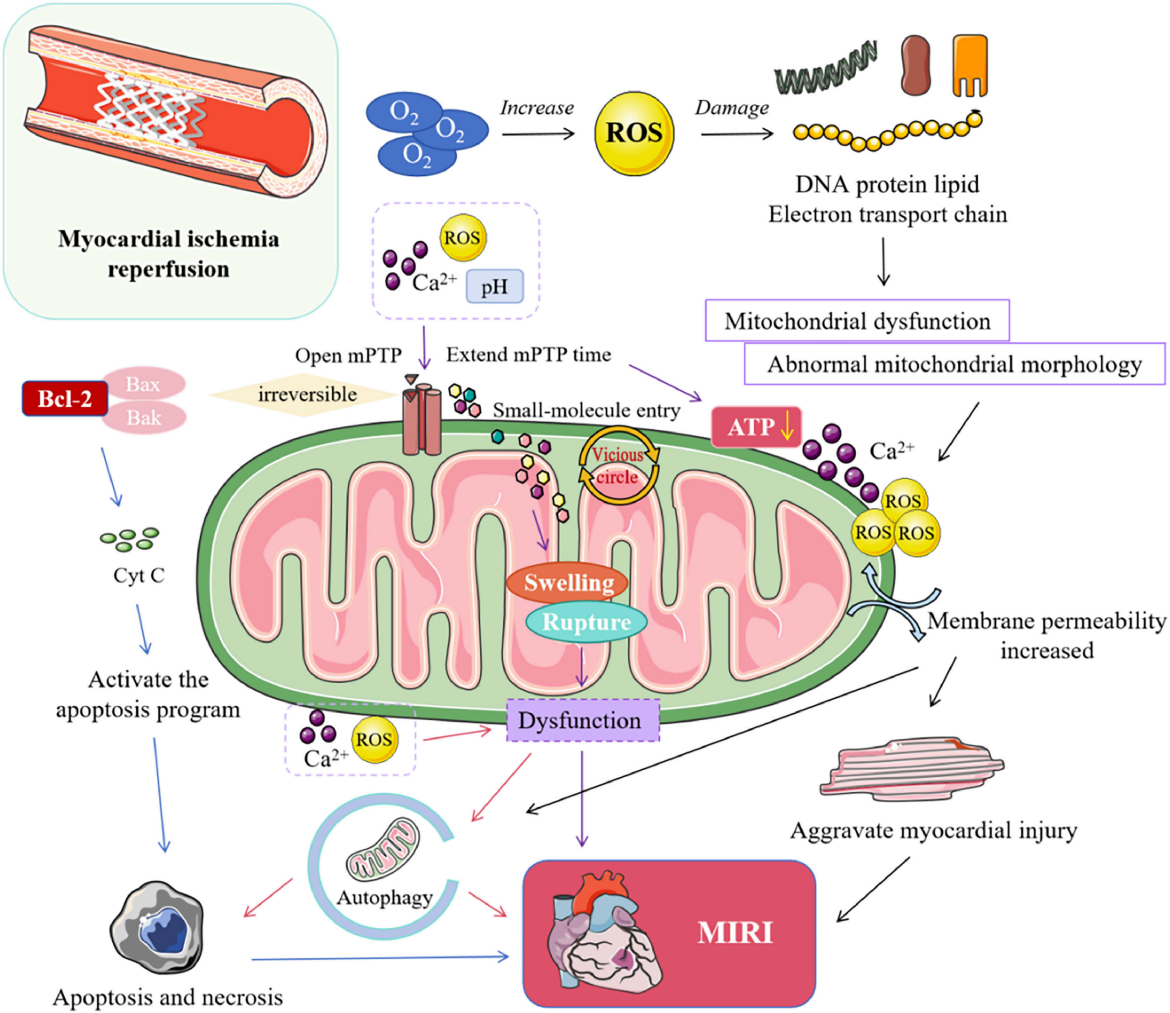
Mitochondrial dysfunction as a core mechanism in MIRI pathophysiology. Reproduced with permission [136]. Copyright 2024, Elsevier GmbH.

Calcium ions (Ca^2+^) are vital regulators of myocardial excitation–contraction coupling and intracellular signaling pathways. However, pathological Ca^2+^ overload during MIRI propagates self-amplifying injury through interdependent mechanisms involving oxidative stress, mitochondrial dysfunction, and inflammation. Reperfusion-phase Ca^2+^ overload primarily originates from dysregulated transmembrane transport mechanisms. Ischemic adenosine triphosphate (ATP) depletion impairs Na^+^/K^+^-ATPase (adenosine triphosphatase) function, elevating intracellular Na^+^ that drives reverse-mode Na^+^/Ca^2+^ exchanger (NCX) activity—the primary Ca^2+^ influx pathway during early reperfusion. Simultaneously, compensatory anaerobic glycolysis generates intracellular acidosis, establishing a proton gradient across sarcolemmal membranes during reperfusion. This proton gradient activates Na^+^/H^+^ exchangers, secondarily elevating cytosolic Na^+^ that further stimulates NCX-mediated Ca^2+^ influx (Fig. 2). In addition, protein kinase C-mediated phosphorylation enhances L-type calcium channel permeability and ryanodine receptor sensitivity, amplifying Ca^2+^ entry [21]. Supraphysiological Ca^2+^ levels activate calpain, causing sarcolemmal disruption and contractile protein degradation, which further promotes intracellular ROS release. Besides, Ca^2+^ accumulation precipitates mPTP opening, disrupting oxidative phosphorylation and activating Bax-dependent apoptosis [22].

### Ferroptosis

Ferroptosis, distinct from traditional programmed cell death pathways (apoptosis, necrosis, and autophagy), was formally termed by Dixon et al. [23] in 2012 as an iron-dependent regulated cell death mechanism. Morphologically, ferroptotic cells exhibit mitochondrial shrinkage with increased membrane density, disorganized cristae structure, and reduced cristae count while maintaining intact plasma/nuclear membranes and uncondensed chromatin [24]. Ferroptosis pathogenesis is primarily driven by dysregulated iron homeostasis, impaired antioxidant amino acid metabolism, and excessive lipid peroxide accumulation [25]. It is involved in the development of various diseases, including MIRI. During myocardial ischemia–hypoxia, metabolic acidosis promotes intracellular reduction of Fe^3+^ to unstable Fe^2+^ ions. These Fe^2+^ ions generate hydroxyl radicals via Fenton reactions, which oxidize polyunsaturated fatty acids (PUFAs) in cellular membranes, resulting in lethal lipid ROS accumulation [26]. Importantly, this pathological cascade is markedly amplified by preexisting oxidative stress and mitochondrial dysfunction in MIRI. Furthermore, ferroptosis both originates from and exacerbates mitochondrial dysfunction, establishing a vicious cycle. GPX4—a key antioxidant enzyme—critically suppresses ferroptosis by preventing oxidative membrane damage. However, MIRI down-regulates GPX4 expression and augments ROS generation. Fang et al. [27] demonstrated that liproxstatin-1 administration during reperfusion elevates mitochondrial GPX4 levels, decreases ROS generation, and consequently attenuates infarct expansion while preserving mitochondrial integrity in murine hearts. Additionally, ischemic cardiomyocytes initiate phospholipid-PUFA redox cycling, driving progressive lipid peroxide accumulation that culminates in membrane disintegration.

### Mitochondrial dysfunction

As illustrated in Fig. 2, mitochondria act as pivotal regulators in MIRI pathophysiology, coordinating responses to oxidative stress and Ca^2+^ overload that determine apoptotic/necrotic pathways. During ischemia, hypoxia directly impairs mitochondrial oxidative phosphorylation, causing ATP depletion, acidosis, and initial intracellular Ca^2+^ accumulation. Upon reperfusion, restored oxygen supply induces mitochondrial electron transport chain dysfunction, triggering ROS overproduction. Concurrently, rapid acidosis correction activates Na^+^/H^+^ exchangers and reverse-mode NCX, causing catastrophic Ca^2+^ overload. Elevated Ca^2+^ and ROS synergistically induce sustained opening of mPTP—nonselective high-conductance channels in the inner mitochondrial membrane—during reperfusion [28]. mPTP opening triggers pathological cascades including mitochondrial depolarization, permeability alteration, energy depletion, and pro-apoptotic factor release (e.g., cytochrome c), culminating in apoptotic and necrotic cell death [29]. Additionally, DAMPs released from damaged mitochondria amplify inflammatory responses. Thus, mitochondria are central to MIRI pathogenesis: initiating energy crises during ischemia, mediating ROS bursts and Ca^2+^ overload during reperfusion, and ultimately driving cell death cascades via mPTP opening [30]. Zhang et al. [31] demonstrated that mitochondrial-targeted delivery of cyclosporine A—a canonical mPTP inhibitor—markedly mitigated MIRI in rat models.

### Microvascular obstruction

Microvascular obstruction is the central pathological mechanism underlying no-reflow phenomenon in MIRI, severely limiting myocardial salvage and increasing long-term adverse outcomes [32]. Major clinical trials indicate that microvascular obstruction affects 60% to 70% of PCI patients, substantially impeding clinical recovery. Ischemic microvascular endothelial damage initiates the process: hypoxia causes endothelial swelling, basement membrane exposure, and platelet/endothelial activation, up-regulating adhesion molecules (P-selectin, ICAM-1). Upon reperfusion, damaged endothelium recruits activated neutrophils and platelets, forming microthrombi that obstruct capillaries. Concurrently, infiltrating leukocytes release proteases and ROS, compromising endothelial barrier integrity and causing plasma extravasation, interstitial edema, and microvascular compression [33]. Endothelial dysfunction disrupts vasomotor balance: Increased vasoconstrictor release (e.g., endothelin-1) and decreased vasodilator synthesis (e.g., NO) cause persistent microvascular spasm. Additionally, reperfusion-shed plaque/thrombus fragments cause distal embolization, and edema-induced cellular swelling mechanically compresses microvessels [34]. Collectively, these processes cause sustained microvascular obstruction (no-reflow). Despite epicardial vessel reopening, myocardial cells remain energy-depleted and progressively damaged due to interrupted oxygen supply, impairing cardiac functional recovery and accelerating adverse ventricular remodeling [35].

## Nanomaterials for MIRI Therapy

### Biomimetic nanomaterials

Recent advances demonstrate cell membrane-coated nanomaterials as promising targeted drug delivery platforms, with growing applications in MIRI therapy (Table 1) [36,37]. These biohybrid nanomaterials preserve synthetic carriers’ physicochemical properties while acquiring native membrane proteins that mediate biological interactions. Membrane modification endows nanomaterials with distinct biofunctional properties depending on donor cell types. Specific examples include the following: (a) Erythrocytic coatings enhance circulation via immune evasion; (b) platelet-derived membranes enable lesion-specific targeting [38]; (c) leukocyte membranes facilitate inflammatory site accumulation [39,40]. This section systematically reviews MIRI therapeutic applications of differentially membrane-engineered nanomaterials.

**Table 1.** Various biomimetic nanomaterials for MIRI

Category	Structure of nanomaterial	Therapeutic agent	Model	Therapeutic effect	Ref.
Macrophages	Pd@CeO_2_-M	Pd@CeO_2_	Mouse	Scavenge ROS	[46]
Macrophages	NA@MEV	MitoN	Mouse	Target the mitochondrial to eliminate the ROS	[48]
Monocytes	Mon-Exo	MSC-exosomes	Mouse	Promote endothelial maturation and modulate macrophage subpopulations	[49]
Macrophages	M2_EV_	/	Rat and pig	Regulate CCR2^+^ macrophages and promote neovascularization	[50]
Macrophages	MMNP_miR199a-3p_	miR-199a-3p	Mouse	Prevent hypoxia-induced apoptosis and promote cell proliferation	[53]
Macrophages	MM-LDH@miR-182	miR-182	Mouse	Inhibited cardiomyocyte pyroptosis, mitigated myocardial fibrosis	[137]
Neutrophils	NM-NP_IL-5_	IL-5	Mouse	Promoted EOS accumulation and angiogenesis	[56]
Neutrophils	CST@CMEV	CST	Mouse	Improve cardiomyocyte apoptosis, excessive fibrosis, macrophage polarization, and angiogenesis	[40]
Neutrophils	Neu-LPs	/	Mouse	Neutralize inflammatory cytokines, modulate inflammatory responses	[57]
Neutrophils	FNLM-miR	miR Combo	Mouse	Induce reprogramming CFs into induced cardiomyocyte-like cells	[58]
Neutrophils	NM@miR	miR-10b	Mouse	Reduce the activation of Hippo-YAP pathway	[59]
Platelets	PNV-CSCs	CSCs	Rat and pig	Increase retention in the heart and reduce infarct size	[67]
Platelets	CS-PGE2-PINC	CSC secretome	Mouse	Augment cardiac function and mitigate heart remodeling	[68]
Platelets	P-EVs	miRs	Mouse	Facilitated the M1 macrophage reprogramming to M2 macrophages	[69]
Platelets	B-P@PLT	BNN6	Mouse	Continuously release NO and increase the local concentration of NO	[70]
Platelets	PLM-miRs	miR-21	Mouse	Realize the reparative reprogramming of the inflamed macrophages	[103]
Platelets	BBR@PLGA@PLT	Berberin	Rat	Promote M2 polarization in macrophages in the early MI phase	[138]
Macrophages + Platelets	BSPC@HM	Sav1 siRNA	Rat and pig	Down-regulate Sav1, interrupt Hippo signaling to foster regeneration	[74]
Erythrocytes + Platelets	Hynocell	hySF	Mouse	Target ischemic tissues and promote vascular regeneration	[75]
Macrophages + Neutrophils	HM4oRL	4-Octyl itaconate	Mouse	Inhibit cardiomyocyte pyroptosis and reprogramming inflammatory signaling	[76]
Exosomes	Exo-I-S	Sirtuin3, GPI-insulin	Rat	Enhances delivery efficiency, improved mitochondrial function and glucose metabolism	[78]
Exosomes	TSA-MSC_exo_	miR-223-5p	Rat	Inhibit CCR2 activation and enhance angiogenesis	[79]
Exosomes	IMTP-MEs-miR-146a	miR-146a	Rat	Myocardium-targeted delivery and potent suppression of inflammatory responses	[80]
Exosomes	KLF2-EXO	miR-486-5p	Mouse	Restrain myocyte apoptosis by targeting the PTEN-PI3K/Akt pathway	[139]
Exosomes	MSC-Exo	miR-199	Mouse	Modulating neutrophil infiltration and NET formation	[140]

#### Monocyte/macrophage membrane biomimetic nanomaterials

Monocyte-derived macrophages serve as key mediators in maintaining immune homeostasis and orchestrating tissue repair processes. Following MIRI, circulating monocytes infiltrate damaged myocardium where they differentiate into effector macrophages [12,41]. During acute inflammation, infiltrating macrophages localize to peri-infarct zones, where they mediate extracellular matrix remodeling and clear cellular debris [42]. The resolution phase commences with declining inflammatory mediator levels, facilitating tissue repair [43]. This transition involves macrophage polarization from pro-inflammatory (M1) to reparative (M2) phenotypes [44]. Macrophage-mimetic nanoplatforms exploit selectin-mediated endothelial interactions to enable inflammation-specific drug targeting [45]. Applying surface engineering and biomimetic principles from materials science, Li et al. [46] engineered Pd@CeO_2_-M nanoparticles by coating palladium/ceria (Pd/CeO₂) cores with macrophage-derived extracellular vesicles (EVs) expressing Mac-1/CD44, demonstrating precise targeting to P-selectin/ICAM-1-rich endothelia via ligand–receptor interactions. Preclinical studies revealed sustained (>24 h) myocardial targeting efficacy of Pd@CeO_2_-M in ischemic models. The nanotherapeutic concurrently inhibits apoptosis/inflammation through multi-pathway modulation, reprograms immune cell trafficking, and stimulates angiogenesis to enhance cardiac functional recovery.

While mesenchymal stem cell (MSC)-derived EVs demonstrate cardioprotective and immunomodulatory properties, their therapeutic potential is constrained by poor tissue homing capacity [47]. Zhang et al. [48] engineered biomimetic nanomotors through MSC-EV/macrophage membrane fusion, enabling deep myocardial delivery of ROS scavengers for cardiac regeneration. Through monocyte membrane functionalization, Zhang’s team [49] enhanced MSC-EV targeting specificity to ischemic myocardium, achieving ameliorating cardiac functional recovery in murine MIRI models.

Li et al. [50] further demonstrated that injecting small EVs derived from M2 macrophages (M2_EV_) into the myocardium of rats and pigs rescues cardiac function, reduces injury markers, and decreases infarct size. Mechanistically, M2_EV_ regulates CC chemokine receptor 2 (CCR2^+^) macrophages via microRNA 181b-5p (miR181b-5p)-dependent mechanisms. This process inhibits CCR2^+^ macrophage recruitment to damaged myocardium and prevents macrophage imbalance.

**Fig. 3.**
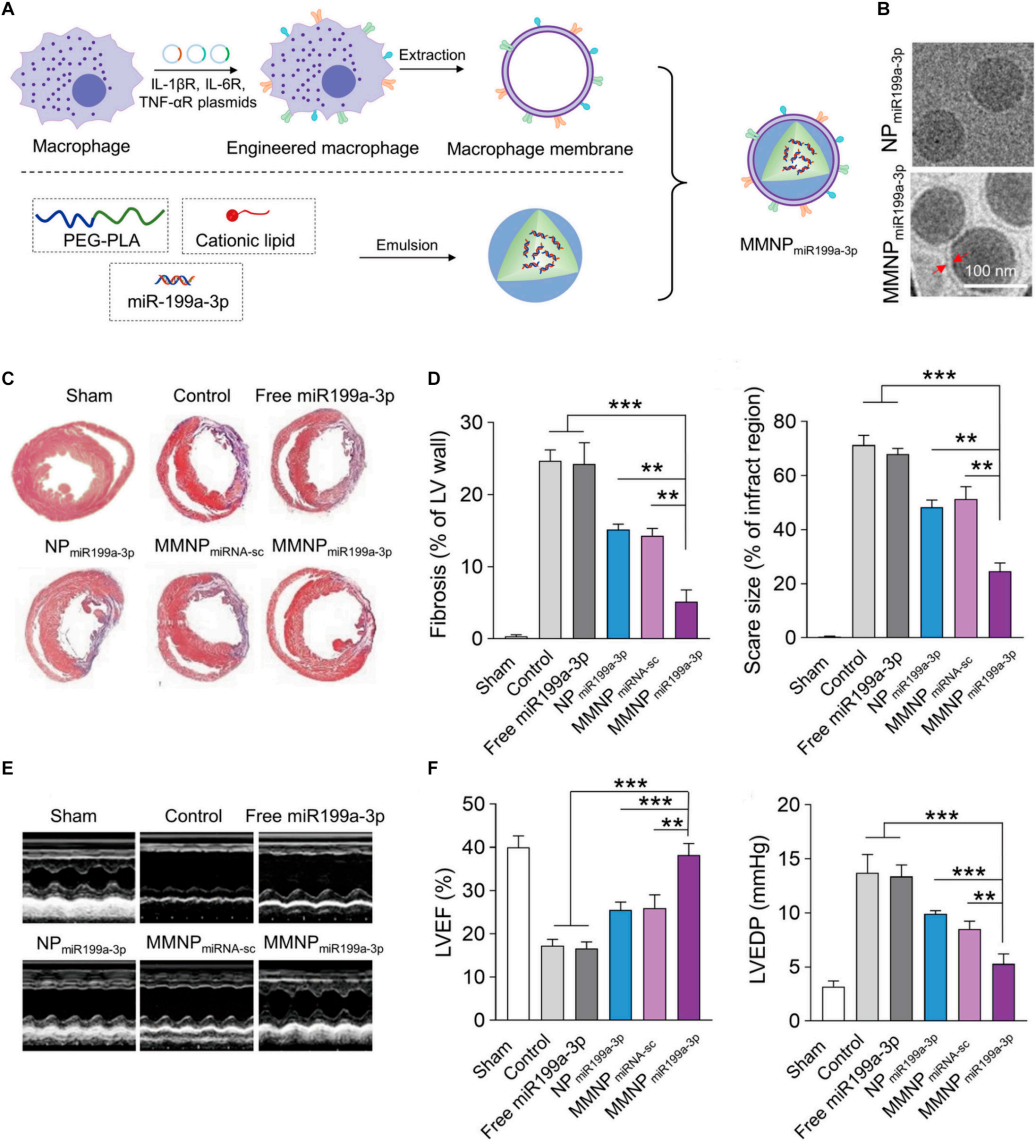
(A) Schematic representation of engineered macrophage membrane-enveloped nanoparticles encapsulating miR199a-3p. (B) Representative images of NP_miR199a-3p_ and MMNP_miR199a-3p_ examined with transmission electron microscopy (TEM). (C) Representative Masson’s trichrome-stained infarcted hearts 2 weeks after treatment (blue, scar tissue; red, viable myocardium). (D) Fibrosis area and scar size of the left ventricular (LV) wall 1 week after treatment. (E) Representative M-mode images 2 weeks after treatment. (F) Cardiac function of mice after different treatments. Reproduced with permission [53]. Copyright 2020, The Authors.

Accumulating evidence from multiple studies demonstrates that miR199a-3p enhances cardiomyocyte proliferation/regeneration and ameliorate cardiac function [51]. Although cationic liposomes effectively penetrate cell membranes as conventional gene delivery carriers, their cytotoxic effects limit clinical applicability [52]. To overcome this issue, Xue et al. [53] developed macrophage membrane-coated nanoparticles (MMNP_miR199a-3p_) containing miR199a-3p. These membranes were extracted from engineered macrophages overexpressing TNF-α, IL-1, and IL-6 receptors (Fig. 3). MMNP_miR199a-3p_ demonstrated superior targeting efficiency and miR delivery to cardiomyocytes compared to free miR199a-3p, effectively reducing apoptosis while enhancing proliferation. Furthermore, MMNP_miR199a-3p_ exhibited notable anti-inflammatory and anti-fibrotic effects, improved ventricular remodeling, and enhanced cardiac function, suggesting therapeutic potential for MI.

#### Neutrophil membrane biomimetic nanomaterials

**Fig. 4.**
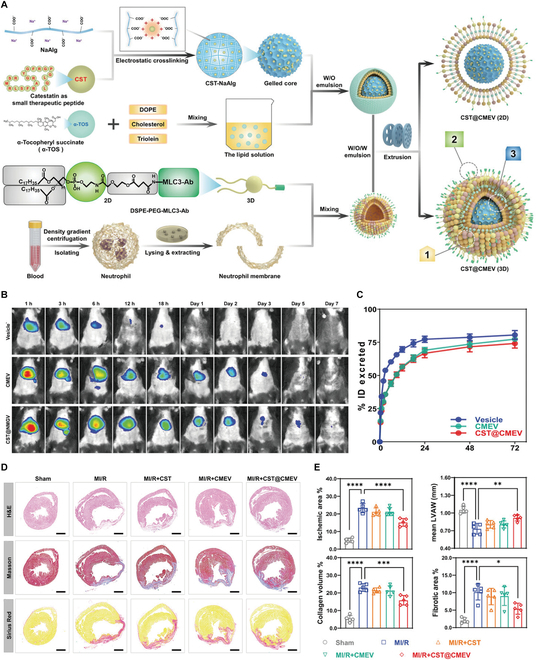
(A) Detailed procedures of CST@CMEV fabrication. (B) In vivo fluorescence imaging of these mice was conducted at different time points. (C) Excretion curve of DiR-labeled materials. (D) Representative images of heart sections stained with hematoxylin and eosin (H&E), Masson, and Sirius Red in the 5 groups. (E) Quantitative analysis of ischemic area, mean thickness of the left ventricular anterior wall (LVAW), collagen volume, and fibrotic area. Reproduced with permission [40]. Copyright 2023, Wiley-VCH GmbH.

Neutrophils, the most abundant leukocytes in peripheral blood, are the first immune cells infiltrating MI sites [41]. They interact with endothelial cells and exhibit temporal dynamics: peaking at 1 to 3 d post-MI before rapid clearance [54]. Che et al. [55] validated neutrophil-mediated delivery of methotrexate-loaded liposomes to myocardial inflammatory sites in MIRI mice. Leveraging neutrophil chemotaxis toward inflammatory signals, researchers have engineered neutrophil-modified nanomaterials for targeted delivery to ischemic myocardium. Han et al. [56] developed neutrophil membrane-encapsulated poly(lactic-co-glycolic acid) (PLGA) cores loaded with IL-5 (NM-NP_IL-5_), effectively addressing the short circulation half-life and delivery limitations of conventional cytokine therapies. By promoting the accumulation of eosinophils and neutralizing multiple inflammatory mediator, NM-NP_IL-5_ can inhibit inflammation-driven cardiomyocyte apoptosis. This combined action attenuated cardiac remodeling and preserved ventricular function. To enhance lesion core targeting, Huang et al. [40] designed a 3-stage targeted nanovesicle (CST@CMEV) combining neutrophil membrane, specific antibody of myosin light chain 3, and catestatin-sodium alginate gelled core (Fig. 4). In vivo and in vitro experiments demonstrated CST@CMEV’s dual functionality: precise lesion core targeting/adherence and sustained catestatin release. This nanomaterial reduced cardiomyocyte apoptosis, attenuated fibrosis, and improved myocardial function in MIRI models.

Current biologics show constrained therapeutic efficacy in MI anti-inflammatory therapy due to 2 key limitations: poor tissue targeting specificity and complex cytokine network interactions. To address these limitations, Chen et al. [57] developed neutrophil membrane-fused liposomes demonstrating dual functionality: inflammatory site homing and cytokine neutralization. This biomimetic system effectively modulates the MI inflammatory microenvironment as a broad-spectrum therapeutic. Similarly, Wang et al. [58] engineered biomimetic membranes combining neutrophil proteins with synthetic lipids, subsequently conjugating FH peptides for tenascin-C targeting. This system achieved cardiac fibroblast (CF)-specific delivery of quadruple miRs for cellular reprogramming. Experimental results demonstrated the system’s ability to enhancing myocardial functional recovery. Building on this platform, the researchers delivered miR-10b through the same targeting mechanism [59]. This treatment promoted cardiomyocyte proliferation via LATS1 inhibition-mediated Hippo pathway modulation.

#### Platelet membrane biomimetic nanomaterials

Platelets originate from bone marrow megakaryocytes and mediate critical biological functions including hemostasis, inflammatory regulation, and tissue regeneration [60]. The platelet membrane contains a unique surface repertoire (GPVI, GPIIb/IIIa, CD47, etc.) that enables dual functionality: enhancing inflammatory targeting through molecular adhesion and prolonging nanoparticle circulation via CD47-mediated immune evasion [61–63]. This biomimetic coating strategy improves nanomaterial biocompatibility

Over the past decade, cell-based therapies have been extensively used in cardiovascular disease treatment [64]. While stem cell therapy shows potential for acute MI treatment, its clinical application is limited by low cell retention and engraftment rates [65,66]. Leveraging platelets’ inherent vascular targeting capability, Tang et al. [67] developed platelet nanovesicle-fused cardiac stem cells (CSCs). This innovative approach enhanced CSC targeting efficiency while maintaining cell viability and functionality in both rat and porcine MI models. Building on this approach, the researchers developed prostaglandin E2 (PGE_2_)-modified platelet membranes to encapsulate PLGA nanoparticles carrying CSC secretome [68]. This design leveraged up-regulated PGE_2_ receptors in ischemic myocardium to achieve targeted therapeutic delivery following MIRI. In vivo studies demonstrated substantially improved cardiac function in MI mouse models treated with platelet-mimicking nanoparticles compared to those receiving CSC transplantation alone.

Li et al. [69] exploited the natural platelet–monocyte interactions following MI to direct platelet membrane-engineered EVs (P-EVs) to ischemic heart tissue. The targeted delivery process initiates when P-EVs bind to Ly-6C^high^-expressing monocytes, which subsequently internalize the vesicles. The miRs released from internalized P-EVs promote macrophage polarization from pro-inflammatory M1 to reparative M2 phenotypes, enhancing cardiac tissue regeneration.

**Fig. 5.**
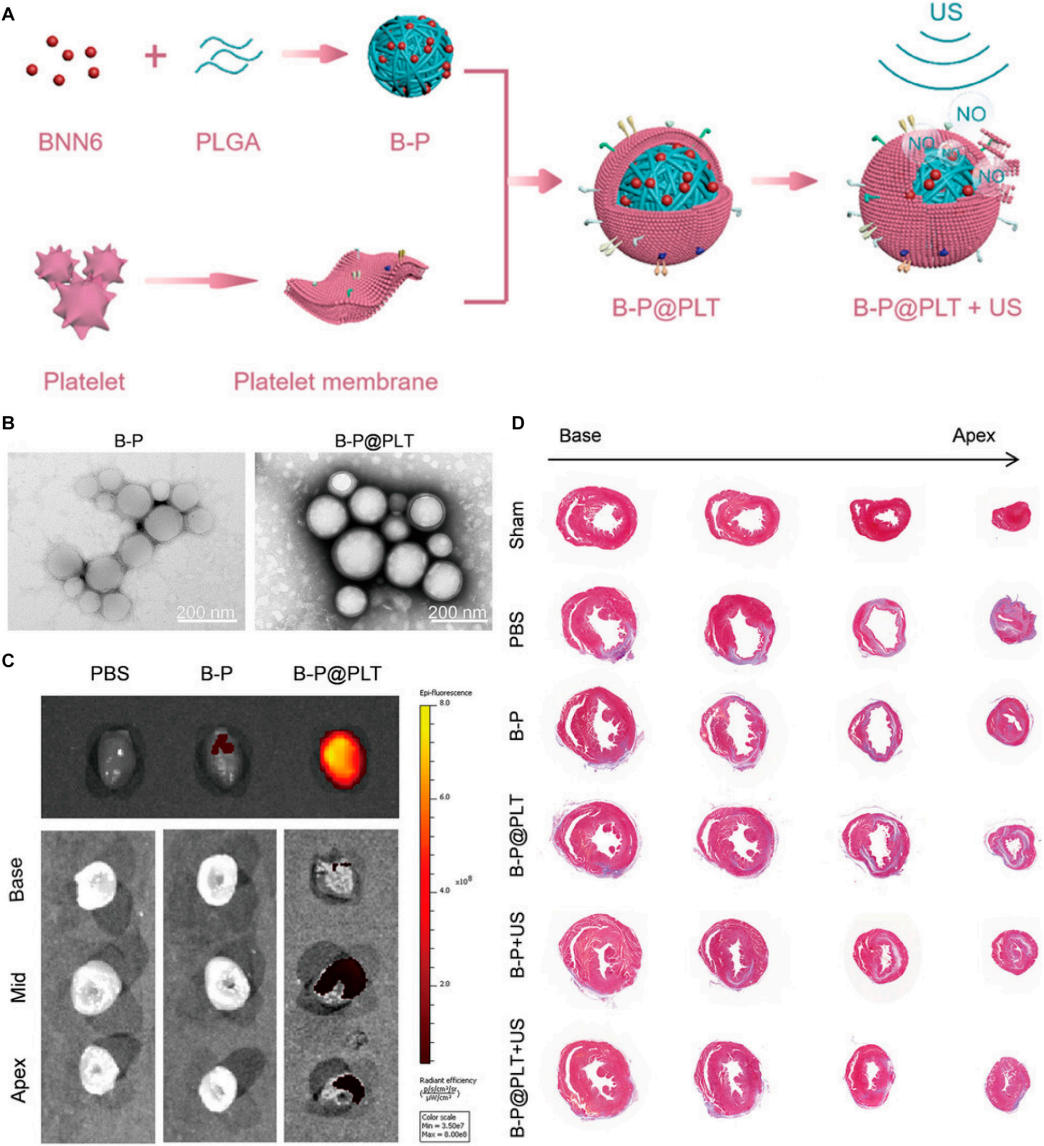
(A) Preparation of B-P@PLT nanoparticles. (B) TEM of B-P and B-P@PLT. (C) Ex vivo optical imaging of the MIRI hearts and the crossing sections from base to apex after intravenous injection with phosphate-buffered saline (PBS), DiR-loaded B-P, or B-P@PLT for 24 h. (D) Masson trichrome staining was performed with 4 paraffin sections evenly separated from cardiac apex to base. Reproduced with permission [70]. Copyright 2023, The Authors.

NO, a crucial signaling molecule in cardiovascular physiology, ameliorates MIRI via multiple molecular pathways. To enable spatiotemporal NO delivery, researchers developed platelet membrane-coated biomimetic nanoparticles (B-P@PLT) incorporating ultrasound-responsive BNN6 NO donors (Fig. 5) [70]. The B-P@PLT system demonstrated 3-fold therapeutic effects: enhancing angiogenesis, preserving cardiomyocyte viability, and attenuating reperfusion-induced myocardial injury upon ultrasound activation.

#### Hybrid membrane biomimetic nanomaterials

**Fig. 6.**
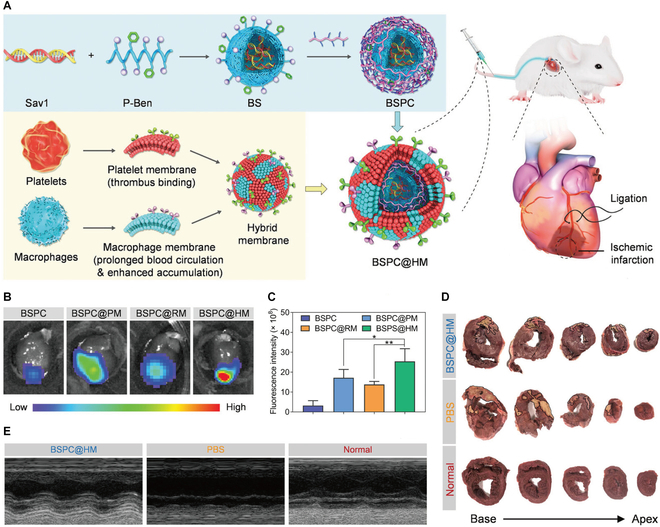
(A) Schematic illustration of the BSPC@HM. (B) Ex vivo imaging and (C) calculated fluorescence intensity of rat hearts at 6 h post-IV injection. (D) Representative 2,3,5-triphenyl-2*H*-tetrazolium chloride (TTC) staining images of heart sections displaying the infarct region. (E) Echocardiographs of pigs. Reproduced with permission [74]. Copyright 2023, Wiley-VCH GmbH.

To address the functional constraints of single-cell membranes, researchers have engineered hybrid membrane (HM) combining distinct cellular characteristics for sophisticated drug delivery applications [71–73]. Zhou et al. [74] developed a dual-membrane system combining platelet-derived thrombus-targeting capability with macrophage membrane-mediated inflammatory homing (Fig. 6). This strategy successfully overcame physiological barriers for myocardial small interfering RNA (siRNA) delivery to ischemic myocardium. In rodent and porcine models, the biomimetic nanomaterials (BSPC@HM) effectively silenced Sav1 gene in damaged myocardium and inhibited its Hippo signal transduction to promote regeneration. Similarly, Zhang et al. [75] engineered erythrocyte–platelet HM-coated PLGA nanoparticles encapsulating hypoxia-preconditioned adipose-derived MSC secreted factors (hySF). This design enhanced ischemic targeting and accelerated angiogenesis. Subsequently, Zheng et al. [76] designed an advanced neutrophil–macrophage HM coating by succinate-preconditioned neutrophil to mimic early MI microenvironments, substantially enhancing damaged myocardium targeting. In murine acute MI models, this integrated platform demonstrates precise targeting of injured myocardium, multidimensional immunomodulation, and substantial cardioprotective efficacy.

Cell membrane coating confers targeting specificity through retained surface receptors (e.g., Mac-1 integrin on macrophage membranes), although performance depends on nanoparticle size and zeta potential. Specifically, neutral/slightly negative surfaces (e.g., platelet membrane-coated NPs) prolong circulation by reducing protein adsorption. It is worth noting that smaller vesicles penetrate fibrotic scars more effectively than larger counterparts.

While cell membrane-coated nanomaterials show promising development in MIRI therapy, critical challenges remain to be addressed. Specifically, platelet membrane-coated nanomaterials carry thrombogenicity risks, while HM modifications have been minimally explored in current research. Systematic investigation of interaction between complex cell membranes and pathological microenvironment is required to develop optimized therapeutic strategies with enhanced safety and efficacy for MIRI management.

#### Exosome-based biomimetic nanomaterials

EVs (30 to 150 nm), particularly exosomes, constitute promising endogenous nanocarriers for MIRI therapy owing to their inherent cardiac tropism, low immunogenicity, and biological barrier permeability. Native exosomes facilitate cardiac homing via surface integrins (e.g., CD47) and deliver parent cell-derived cargo (including miRs and proteins) to modulate the cardiac immune microenvironment. For instance, MSC-derived exosomes (MSC-Exo) delivering miR-182 attenuate inflammation through TLR4/NF-κB pathway inhibition, thereby reducing infarct size in murine models [47].

To address limitations of natural exosomes—including low drug efficacy and poor targeting—engineering modification strategies have emerged that substantially enhance their therapeutic potential as drug delivery vehicles [77]. Yang et al. [78] genetically engineered MSCs to coexpress Sirtuin3 and glycosylphosphatidylinositol (GPI)-anchored insulin genes, subsequently isolating engineered exosomes (Exo-I-S). This dual payload synergistically enhances delivery efficiency and myocardial retention while alleviating MIRI through improved mitochondrial function and glucose metabolism. Similarly, Li et al. [79] preconditioned MSCs with tanshinone IIA (TSA) to generate miR-223-5p-enriched exosomes (TSA-MSC_exo_). Intramyocardial TSA-MSC_exo_ delivery conferred enhanced cardioprotection in MIRI models versus controls. In Meng’s study, they engineered cardiac-targeted exosomes (IMTP-MEs-miR-146a) through dual modification [80]. Versus unmodified controls, ischemic myocardial targeting peptide-functionalized milk exosomes (MEs) enhanced myocardial miR-146a delivery efficiency, potently suppressed NF-κB signaling via IRAK1/TRAF6 down-regulation, and demonstrated superior cardioprotection post-injection.

Although genetic modification, cellular preconditioning, and surface functionalization effectively enhance exosome targeting, payload delivery, and therapeutic efficacy against MIRI in preclinical models, critical clinical translation barriers remain. Key challenges include limited scalability and reproducibility of complex processes (e.g., electroporation, conjugation, and genetic engineering), immunogenicity risks from heterologous sources like MEs, and insufficient standardized data on long-term safety and comparative efficacy across platforms. Future research should focus on overcoming translational barriers by developing Good Manufacturing Practice (GMP)-compliant protocols and establishing predictive models that assess clinical viability beyond proof-of-concept validation.

#### Lipoprotein-mimetic nanomaterials

Lipoproteins are essential complex particles that transport lipids, particularly cholesterol, throughout the human body. These particles are recognized and internalized via specific cell surface receptors, including the key scavenger receptor BI (SR-B1), which mediates high-density lipoprotein (HDL) uptake, enabling targeted lipid transport [81]. Inspired by this natural targeting mechanism, synthetic lipoprotein nanoparticles precisely mimic the structure and function of natural lipoproteins. These nanoparticles leverage the SR-B1 receptor-mediated pathway to deliver therapeutic drugs specifically to ischemic myocardial tissue. This strategy’s efficacy has been demonstrated in studies of apolipoprotein AI nanoparticles (n-apo AI). Experiments demonstrate that intravenous injection of n-apo AI immediately after MI markedly attenuates systemic and cardiac inflammation, evidenced by reduced circulating leukocytes and diminished cardiac infiltration [82]. Mechanistically, n-apo AI acts directly on ischemic cardiomyocytes and leukocytes—particularly neutrophils and proinflammatory monocytes—via SR-B1 and other receptors. These findings indicate that lipoprotein nanoparticles show promise for MI/RI. However, current research predominantly examines short-term outcomes. Key knowledge gaps persist regarding long-term functional improvement, validation in complex preclinical models, and carrier delivery efficiency within pathological microenvironments post-severe MIRI such as no-reflow zones. Future studies should optimize carriers through enhanced targeting precision, controlled-release capability, and immunogenicity reduction, and should develop multifunctional co-loading platforms for therapeutic agents. Moreover, clinical translation requires comprehensive safety profiling and pharmacokinetic assessment, particularly in complex comorbidities like hyperlipidemia or diabetes. This will advance these bioinspired nanocarriers toward becoming effective interventions for improving post-reperfusion prognosis in MI patients.

### Inorganic nanomaterials

Inorganic nanomaterials, primarily comprising metallic and nonmetallic elements, allow precise structural customization for specific biomedical applications [83]. These nanomaterials feature nanoscale dimensions and high surface-to-volume ratios. Furthermore, they exhibit unique physicochemical properties (e.g., optical, electrical, and magnetic characteristics) with applications spanning drug delivery, bioimaging, and photothermal therapy [72,84].

#### Metallic nanomaterials

Metallic nanomaterials are nanoscale solid particles composed of metallic elements including gold, silver, iron, copper, and platinum [85]. Precise control of size, morphology, compositional gradients, and surface functionalization enables engineering of multifunctional metallic nanomaterials [86]. These nanomaterials demonstrate distinct functionalities: Platinum/palladium-based systems exhibit catalytic and antioxidant properties [46,87], gold nanostructures display exceptional conductivity [88], while iron oxide variants possess superparamagnetic behavior [89,90]. Leveraging these unique properties, researchers have developed tailored metallic nanomaterial platforms for MIRI therapy (Table 2).

**Table 2.** Representative metallic nanomaterials for MIRI

Category	Structure of nanomaterials	Materials for decoration	Model	Properties	Ref.
Pt atoms	PtsaN-C	/	Mouse	Multiple enzyme-mimicking activities for ROS scavenging	[87]
MnO_2_	Mito-fenozyme	TPP	Mouse	SOD- and CAT-like activities and mitochondrial targeting ability	[92]
Fe^3+^	Fe-Cur@TA	TA	Mouse and beagle dog	Excellent free radicals scavenging and anti-inflammatory properties	[93]
Ce	TA-Ce NCs	TA	Mouse	Myocardial targeting and elimination of oxidative substances	[96]
Fe_3_O_4_	GMNP_EC_	Anti-CD63 and anti-MLC	Rabbit and rat	Collect, transport, and release circulating exosomes to the infarction area	[89]
Mn_3_O_4_	Mn_3_O_4_@PDA-MSCs	PDA	Mouse	Efficient SOD mimic and excellent MRI contrast agent for tracking MSCs	[97]
Gold nanorods	AS-I/S NCs	IMTP and SS31	Rat	Enhanced antioxidant and real-time photoacoustic tracking imaging	[98]
Gold nanocages	L-Arg@Au@Se@PCM	PCM	Rat	Enhanced ROS inhibition and NO generation ability	[99]
Sr	SrCO_3_/HSA	/	Mouse	Stimulate angiogenesis and maintain cardiomyocyte viability	[141]
Fe_3_O_4_	IONP-NVs	/	Rat	Enhance retention and improve therapeutic molecular level	[90]
Cerium	CVNRs	/	Rat	Considerable ROS scavenging and cell-protecting activity	[94]
Fe^3+^, Fe^2+^	PBNz@PSC	PSC	Mouse	Multiple enzyme-like activities and myocardial targeting ability	[142]
Cu, Mn	Cu-TCPP-Mn	/	Mouse and rat	SOD- and CAT-like activities to achieve ROS-scavenging effect	[143]
Ru	RuO2@BSA	/	Mouse	Prominent SOD- and CAT-like performance	[144]
Zn, Au	miR-30d@ZIF-8^+^ AuNPs	/	Mouse	Localized/sustained miRNA delivery to infarcted myocardium	[145]

**Fig. 7.**
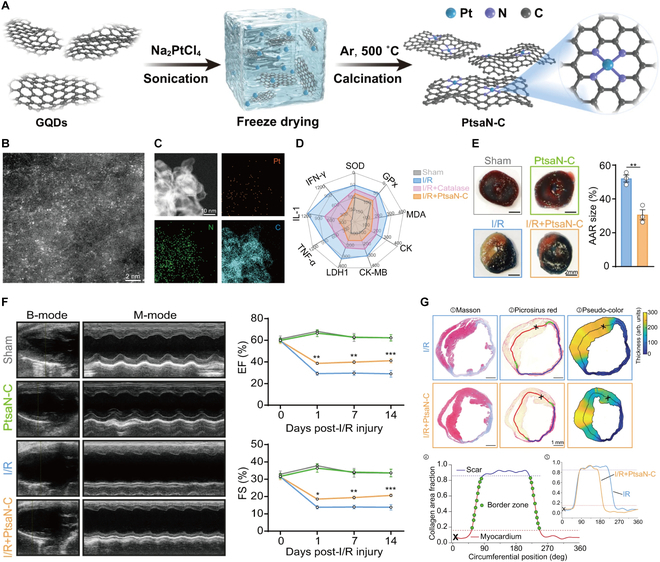
(A) Schematic illustration of the preparation strategy for PtsaN-C. (B) High-angle annular dark field scanning TEM image of PtsaN-C, where the bright dots correspond to Pt single atoms. (C) Energy-dispersive spectrometer mapping images of PtsaN-C. (D) Radar chart plot of the indicated indexes. (E) Representative photographs of Evans blue/TTC double staining and the corresponding quantified results. (F) Cardiac function detected by echocardiography. (G) Pipeline analysis of scar thickness, infarct size, and border zone transition. Reproduced with permission [87]. Copyright 2024, The Authors.

Overproduction of ROS in infarcted myocardium induces oxidative stress and mitochondrial dysfunction. Endogenous antioxidants [e.g., superoxide dismutase (SOD), GPX, and catalase (CAT)] exhibit limited ROS-scavenging capacity in pathological conditions. Nanozymes are a class of artificial enzymes that combine nanomaterial properties with enzyme-like catalysis [91]. Their design fundamentally relies on materials science principles. These nanomaterials address inherent limitations of natural enzymes (e.g., structural instability, low production yield, and demanding storage requirements), emerging as promising therapeutic agents for MIRI [92–95]. Nanozymes efficacy is modulated by size and surface properties: Single-atom structures can maximize atomic utilization and improve ROS-scavenging efficiency, while larger particles are conducive to magnetic residence. Positively charged surfaces enhance mitochondrial targeting but accelerate hepatic clearance by the reticuloendothelial system, necessitating optimized design to balance targeting and pharmacokinetics. Leveraging the inherent stability and biocompatibility of platinum (Pt), Ye et al. [87] engineered single-atom Pt nanozymes with Pt-N_4_-C coordination (PtsaN-C) for targeted MIRI therapy (Fig. 7). The PtsaN-C system exhibits multi-enzyme mimetic activity, enabling efficient ROS scavenging that substantially attenuates myocardial damage and reduces infarct area.

**Fig. 8.**
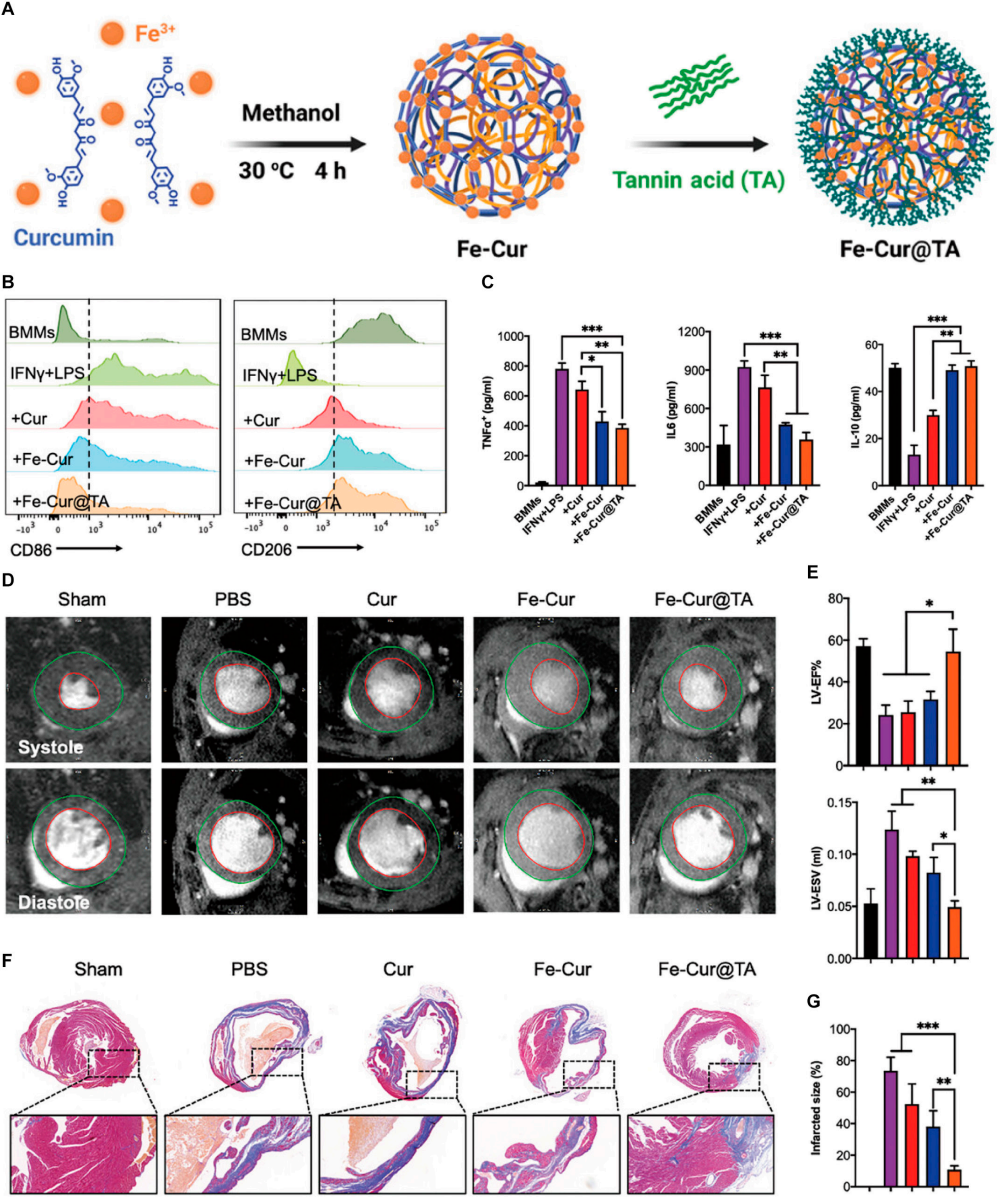
(A) Schematic illustration of preparation of drug-based nanozyme (named as Fe-Cur@TA). (B) Representative flow cytometry data of activated bone marrow macrophages after stimulation with different treatments and quantification of M1 (CD86) and M2 (CD206) macrophages. (C) Levels of cytokines after stimulation in the various experimental groups. (D and E) Representative MRI images and cardiac function of MI mice measured on day 12 after various treatments. (F and G) Representative Masson’s trichrome images and the quantification of the size of the cardiac infarcts. Reproduced with permission [93]. Copyright 2023, Wiley-VCH GmbH.

Since mitochondria are a primary site of ROS production, mitochondrial targeting of therapeutic drugs is critical to enhance efficacy. Zhang et al. [92] developed Mito-Fenozyme, a biomimetic cascade nanozyme using MnO_2_ as the enzymatic core conjugated with mitochondria-targeting triphenylphosphonium (TPP). This nanozyme exhibits dual SOD- and CAT-mimetic activities, enabling sequential conversion of O_2_^•−^ to H_2_O and O_2_ to enhance cardiac functional recovery. However, clinical translation remains challenged by insufficient cardiac-specific targeting. Therefore, developing strategies to effectively target the infarcted heart and enhance local drug retention remains a critical challenge requiring further investigation. Tannic acid (TA)-based surface modification has emerged as a promising myocardial targeting strategy [6]. Liu et al. [93] engineered TA-modified metallic nanozymes by coordinating Fe^3+^ with curcumin, achieving enhanced myocardial accumulation. Both in vitro and in vivo studies demonstrated its free radical scavenging capacity through CAT/SOD-mimetic activities, effectively preserving mitochondrial integrity. Application in MI animal models induced macrophage polarization from pro-inflammatory MI to reparative M2 phenotypes, up-regulating Arg1 and IL-10 while suppressing inflammatory mediators (Fig. 8). Similarly, in another study, TA coordinated with tetravalent cerium ions to self-assemble into myocardial-protective nanocatalysts (TA-Ce NCs) [96].

Unlike conventional targeting approaches, Liu et al. [89] engineered a magnetite (Fe_3_O_4_) core with inherent magnetic targeting properties. This nanoparticle was encapsulated in poly (ethylene glycol)-modified silica and functionalized with dual-targeting antibodies: anti-CD63 (specific to exosomal antigens) and anti-myosin light chain (targeting damaged cardiomyocytes in MI regions), enabling precise delivery of circulating exosomes to infarcted areas for therapeutic intervention.

**Fig. 9.**
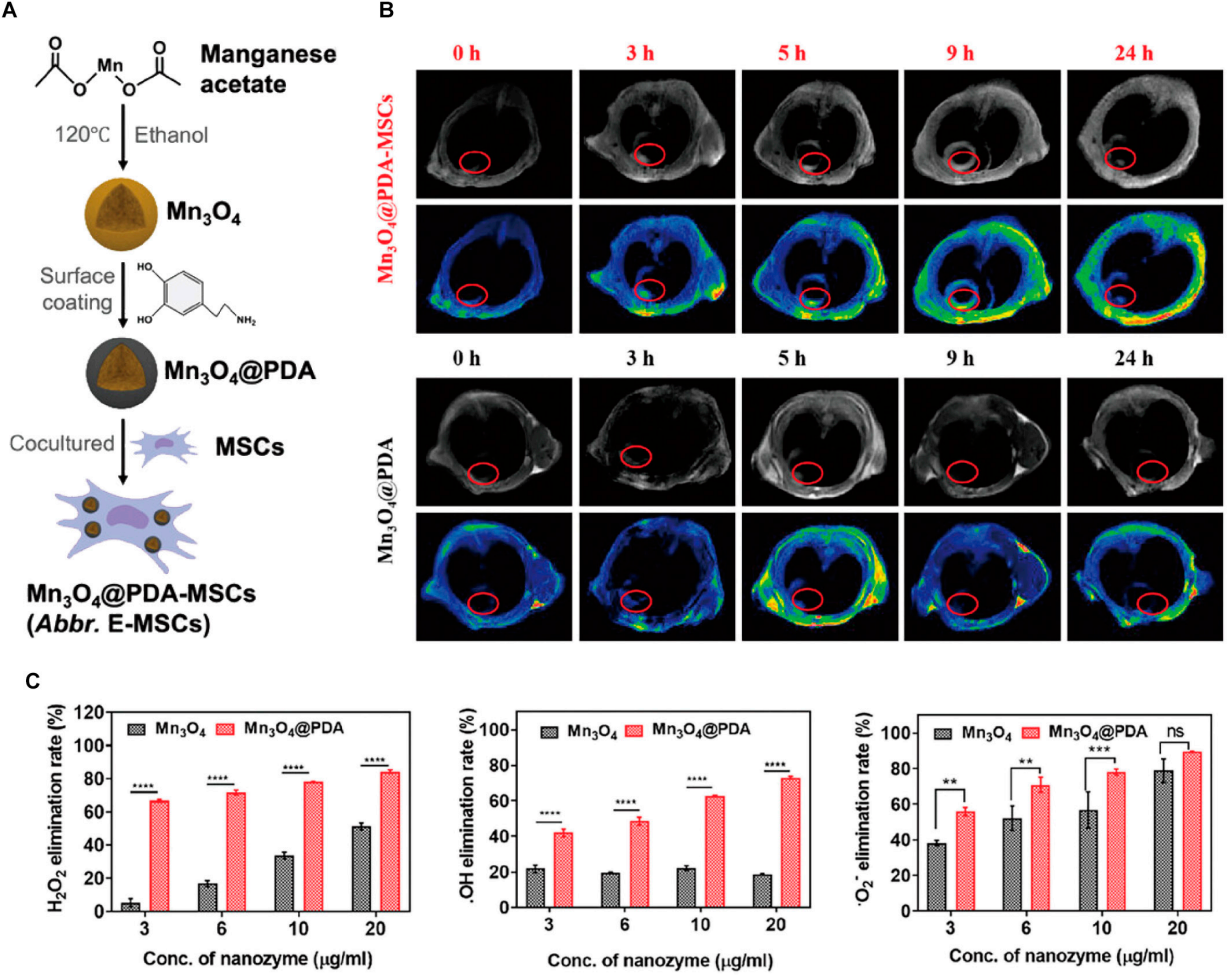
(A) Schematic illustration of the preparation of E-MSCs. (B) Time-dependent MRI of E-MSCs and Mn_3_O_4_@PDA at heart sites in MI mice. (C) ROS-scavenging activity of Mn_3_O_4_ and Mn_3_O_4_@PDA. Reproduced with permission [97]. Copyright 2024, Wiley-VCH GmbH.

Nanozymes possess inherent multimodal imaging capabilities due to their unique optical, magnetic, and acoustic properties, complementing their therapeutic functions in antioxidant delivery and drug transport. Building on these properties, Le et al. [97] engineered a theranostic nanozyme by encapsulating Mn_3_O_4_ with polydopamine (PDA) (Fig. 9). The nanozyme was internalized by MSCs via endocytosis, generating functional E-MSCs (Mn_3_O_4_@PDA-MSCs). In MI models, E-MSCs demonstrated dual therapeutic and diagnostic functions: ROS scavenging for cardiac repair and magnetic resonance imaging (MRI)-guided tracking of cell localization and injury mapping.

**Fig. 10.**
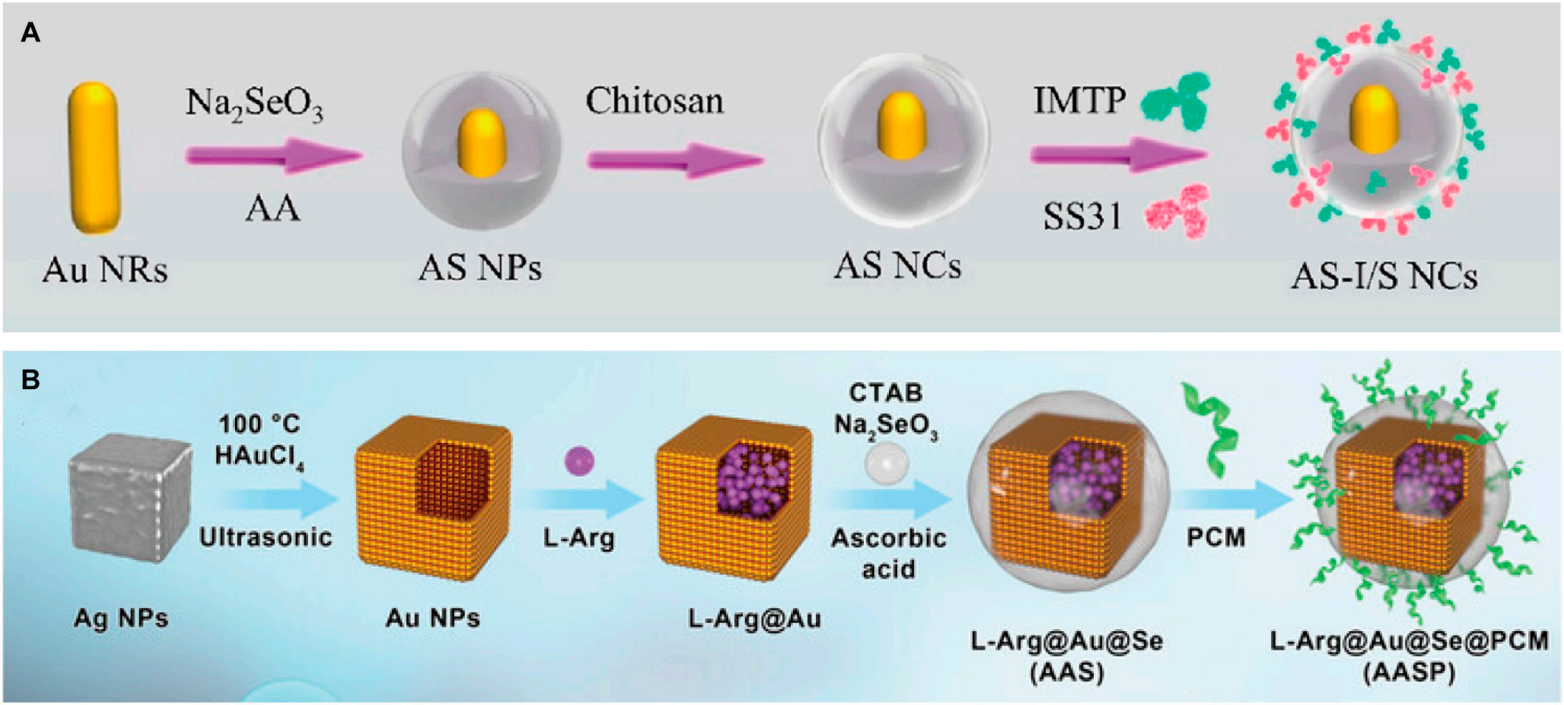
(A) Schematic illustration of the fabrication of the AS-I/S NCs. Reproduced with permission [98]. Copyright 2022, American Chemical Society. (B) Synthesis route of L-Arg@Au@Se@PCM. Reproduced with permission [99]. Copyright 2023, The Authors.

In a pioneering approach, researchers engineered near-infrared II (NIR-II)-responsive gold nanorods as the structural core. These nanomaterials were sequentially encapsulated with selenium nanoparticles (Se NPs) and chitosan and then surface-functionalized with dual-targeting motifs: ischemic myocardial targeting peptide (IMTP) and mitochondrial-directed antioxidant peptide SS31, ultimately establishing an integrated imaging-therapeutic system for MIRI (Fig. 10A) [98]. Post-administration, the system demonstrated enhanced ischemic myocardium targeting efficiency with prolonged retention, markedly improving cardiac function while suppressing key myocardial injury biomarkers. Building on this concept, Wang et al. [99] developed hollow gold nanocages encapsulated with Se NPs, which were further conjugated with cardiomyocyte-targeting peptide (CMP) PCM to enable precision delivery of L-arginine for MIRI rehabilitation (Fig. 10B). These studies highlight the potential of metallic nanomaterials not only for therapeutic intervention but also for real-time imaging guidance and monitoring of myocardial injury, as demonstrated by complementary techniques like hemispherical photoacoustic imaging [100].

Metallic nanomaterials demonstrate multifunctional integration of antioxidant, conductive, and imaging properties (optical/magnetic), positioning them as promising candidates for MIRI therapy. However, clinical translation still faces challenges, such as iron oxide nanoparticles may disrupt iron homeostasis, paradoxically inducing myocardial oxidative stress and aggravating cardiac injury. The high surface-to-volume ratio of metallic nanomaterials creates unstable surface defects and dangling bonds, leading to particle agglomeration that compromises structural stability and long-term therapeutic efficacy. The long-term biodistribution profiles, chronic toxicity risks (particularly from nonbiodegradable metallic species like Pt and Au), and organ-specific bioaccumulation (e.g., hepatic/splenic retention) require rigorous evaluation in physiologically relevant models. Future research should focus on engineering stable metallic nanostructures with enhanced catalytic activity and tissue-specific targeting capabilities to address current clinical limitations. Resolving these challenges is essential to realize the clinical potential of metallic nanotherapeutics for MIRI therapy.

#### Nonmetallic nanomaterials

Nonmetallic nanomaterials have emerged as key therapeutic-diagnostic agents for MIRI, complementing their metallic counterparts. These materials primarily comprise carbon-based systems (graphene oxide, carbon dots, carbon nanotubes, etc.) and silicon-based architectures [mesoporous silica, silicon nanowires (SiNWs), etc.].

**Fig. 11.**
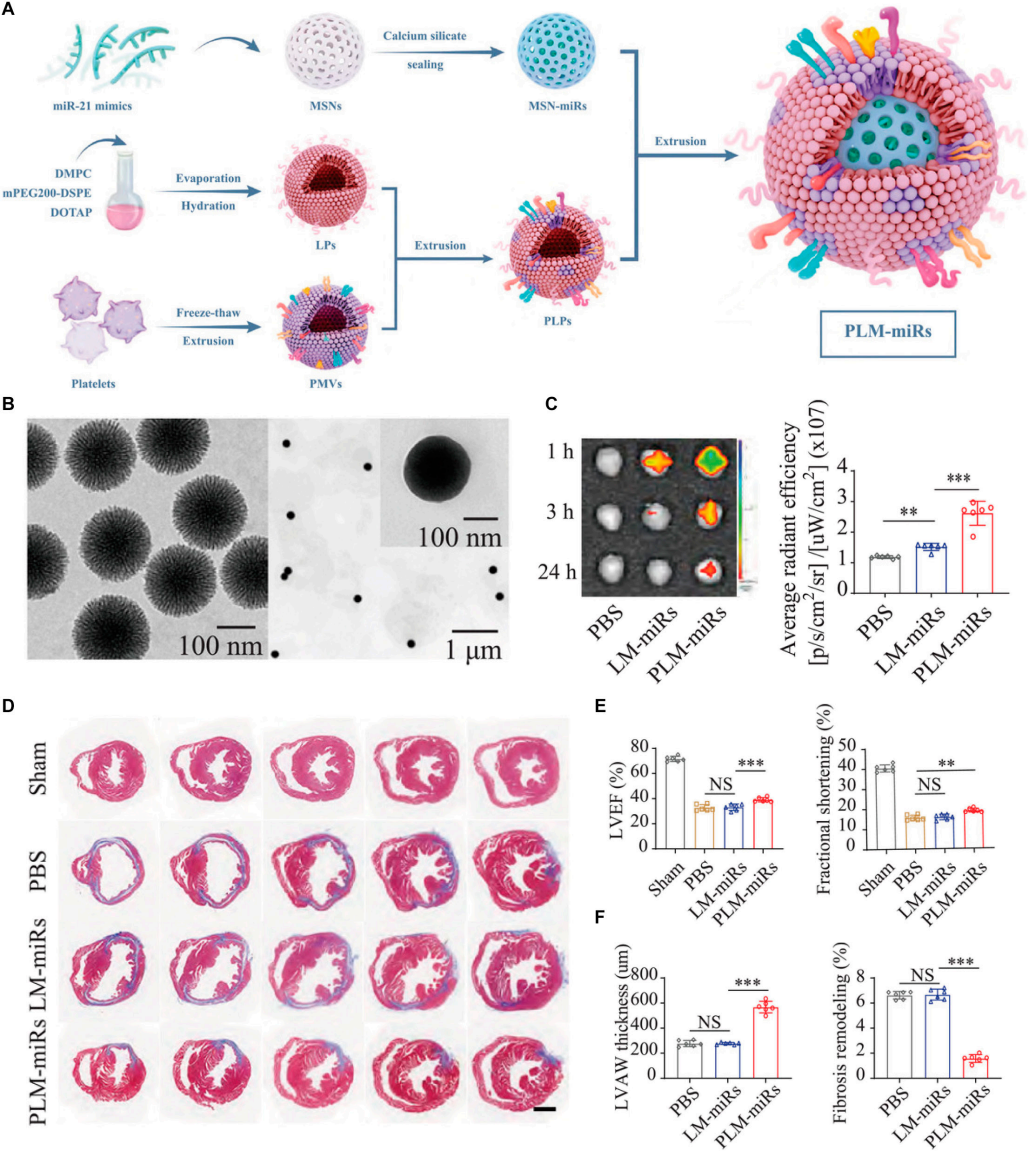
(A) Fabrication of platelet-like fusogenic liposome-coated mesoporous silica nanospheres loaded with miR-21 mimics (PLM-miRs). (B) TEM of MSNs (left) and PLM-miRs (right). (C) In vivo spectrum imaging system images of 3 time points of materials accumulated in the hearts and the data of the first time point were further quantified. (D) Masson staining of MIRI heart paraffin sections at 4 weeks after various treatments. (E) Cardiac function was assessed by echocardiography at 4 weeks after treatment. (F) LVAW thickness and fibrosis remodeling in (D) was quantified by using ImageJ software. Reproduced with permission [103]. Copyright 2021, The Authors.

Mesoporous silica nanoparticle (MSN) demonstrates exceptional characteristics, including high biocompatibility, facile surface modification, large specific surface area, stable porous architecture, and tunable pore dimensions [101,102]. These properties render them ideal carriers for therapeutic agent delivery. Tan et al. [103] engineered a calcium silicate-gated MSN system to load negatively charged miR-21 (termed MSN-miRs), which can effectively prevent miR from being engulfed by lysosomes (Fig. 11). The construct was further functionalized with a biomimetic platelet membrane-cationic liposome hybrid coating, demonstrating enhanced targeting specificity and delivery efficacy. MSNs with pore sizes >5 nm enable high-loading capacity for miR, whereas surface-grafted cationic liposomes enhance monocyte-mediated nanoparticle uptake. These engineered nanoparticles specifically target cardiac-infiltrating monocytes within ischemic myocardium, promoting macrophage repolarization toward an anti-inflammatory phenotype, consequently attenuating MIRI-induced cardiac dysfunction. A high-capacity MSN platform was developed to encapsulate the miR Combo (miR-1, miR-133a, miR-208, and miR-499), which mediates fibroblast-to-cardiomyocyte reprogramming [58]. This protection enhanced the therapeutic efficacy in reducing fibrosis and restoring cardiac function. Beyond miRNA delivery, MSNs serve as a versatile platform for transporting diverse therapeutics targeting MIRI. For example, it can deliver anti-inflammatory dexamethasone to inhibit the NF-κB signaling pathway, thereby working together with the receptor for advanced glycation end product siRNA to exert anti-inflammatory effects [104]. Moreover, the platform has successfully delivered cardioprotective agents including notoginsenoside R1,[105], quercetin [106], and adenosine to MI regions, demonstrating targeted therapeutic effects.

Meanwhile, silicon can also be applied in the form of SiNWs for the treatment of MI. SiNWs demonstrate distinct advantages over conventional conductive nanomaterials, particularly their inherent biocompatibility and tunable electrical properties. Tan et al. [107] engineered SiNW-incorporated cardiac organoids that improved electrophysiological synchronization in transplanted cardiomyocytes. This nanowired platform effectively mitigated adverse ventricular remodeling and promoted functional cardiac recovery.

In addition, nonmetallic nanomaterials demonstrate considerable potential for early detection of MI. Li et al. [108] engineered an ultrasensitive carbon dot-based photoelectrochemical biosensor. This device specifically quantifies glutathione levels in ischemic cardiomyocytes, providing a novel approach for MI severity assessment.

Current research on nonmetallic nanomaterials for MIRI remains limited. Nevertheless, these materials provide critical advantages—notably superior biocompatibility and reduced intrinsic toxicity versus metallic alternatives. Tunable porosity and surface chemistry (e.g., in MSNs) allow controlled release kinetics of therapeutic molecules and enhanced cellular uptake/transfection efficiency. Key limitations include suboptimal biodegradation kinetics in some formulations (e.g., specific silica nanomaterials), which may cause long-term bioaccumulation. Although MSNs exhibit high drug-loading capacity, achieving >80% payload efficiency for nucleic acid therapeutics requires further optimization. Addressing these limitations requires focused development of enhanced biodegradation profiles, optimized payload delivery, and novel nonmetallic formulations—critical for realizing the clinical potential of targeted MIRI nanotherapies.

### Hydrogel

Hydrogels are 3-dimensional polymer networks swollen with water through physical or chemical cross-linking [109]. Cross-linking density governs hydrogel degradation and drug release kinetics: Low-density networks facilitate short-term burst release, whereas high-density configurations enable sustained long-term release. Owing to their soft texture, high water content, and structural resemblance to human soft tissues, hydrogels have become versatile biomaterials suitable for wound healing, tissue repair, and regenerative applications [110,111]. Recent advancements in hydrogel formulations for orthotopic treatment of MI highlight their potential to provide mechanical support and localized drug delivery in ischemic heart tissue [112–114]. Researchers have engineered diverse hydrogel systems tailored to specific pathophysiological mechanisms underlying MI.

Leveraging the pathological hallmark of elevated ROS in infarcted myocardium, Li et al. [115] engineered a ROS-responsive poly(vinyl alcohol) hydrogel network for targeted delivery of basic fibroblast growth factor (bFGF). Upon ROS activation, the hydrogel undergoes controlled degradation, enabling sustained release of bFGF that diffuses into myocardial tissue to promote functional recovery and attenuate fibrotic progression. Similarly, Hao et al. [116] developed an innovative chitosan-based hydrogel functionalized with boronate-protected diazeniumdiolate, achieving concurrent NO release and ROS scavenging to mitigate pathological cardiac remodeling.

**Fig. 12.**
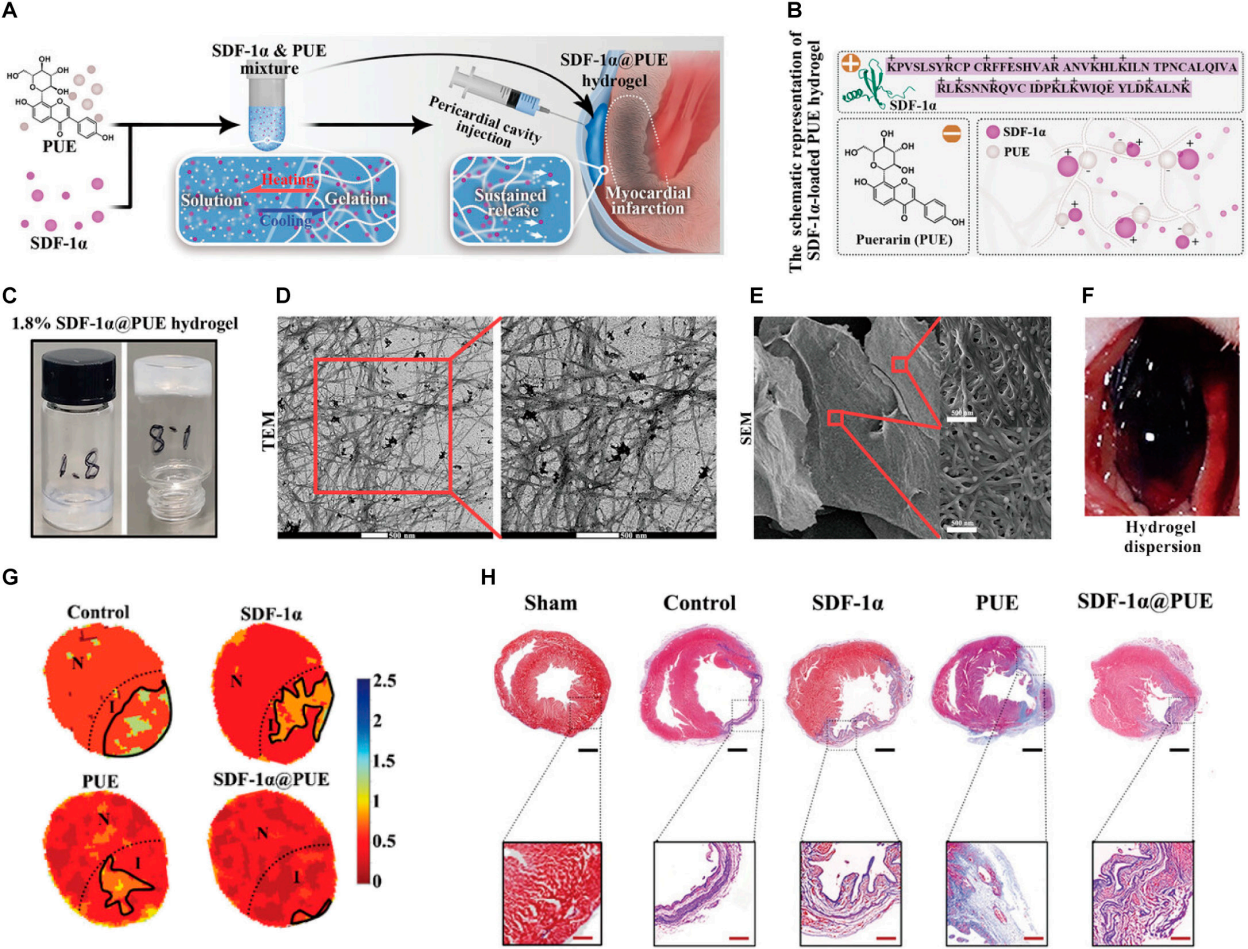
(A) Schematic illustration of SDF-1α@PUE fabrication and intrapericardial injection. (B) SDF-1α@PUE hydrogel formation from PUE and SDF-1α by electrostatic interaction. (C) Hydrogel-forming image of SDF-1α@PUE. (D and E) TEM and scanning electron microscope images of the hydrogel. (F) Dispersion of SDF-1α@PUE hydrogel after intrapericardial injection. (G) Revascularization was assessed using isolated perfused hearts. (H) Masson’s trichrome staining images of infarcted hearts after different treatments for 28 d. Reproduced with permission [118]. Copyright 2023, Wiley-VCH GmbH.

To mitigate inflammatory responses in MI, Lee et al. [117] developed a gelatin–dextran aldehyde hydrogel incorporating angiopoietin-like protein 4 (ANGPTL4), an anti-inflammatory cytokine, for targeted therapy. This adhesive hydrogel provides dual functionality: cardiac surface conformal support and sustained ANGPTL4 release to inhibit maladaptive tissue remodeling. Luo et al. [118] engineered a self-assembling puerarin (PUE) hydrogel that electrostatically binds stromal-derived factor-1α (SDF-1α), creating the SDF-1α@PUE composite for myocardial repair (Fig. 12). The construct modulates the inflammatory niche through enhanced endogenous stem cell homing, while improving electromechanical coupling via optimized signal transduction pathways, collectively enhancing post-MI recovery. As a complementary strategy, a myocardially injected poly (amino acid) hydrogel was developed to simultaneously inhibit apoptosis and inflammation, and to achieve synergistic therapeutic effects [119]. This approach provides a biocompatible platform for localized multi-pathway intervention. Further supporting the role of poly(amino acid) hydrogels in myocardial protection, studies have demonstrated their intrinsic ability to mediate cytoprotection specifically through antioxidant mechanisms [120].

While mitochondrial-derived peptide MOTS-c demonstrates therapeutic potential in regulating MI-induced metabolic dysregulation, its clinical translation is constrained by rapid systemic clearance. To overcome these pharmacokinetic challenges, Zhang et al. [121] developed an injectable hydrogel (termed MQ^gel^) through chemical conjugation of MOTS-c with the self-assembling polypeptide QQKFQKQKEQQ. This formulation can modulate bioenergetic metabolism to restore mitochondrial homeostasis, and augment cardiomyocyte viability.

Post-MI, fibrotic scar tissue displaces functional cardiomyocytes, disrupting electrophysiological signal propagation and precipitating cardiac mechanical dysfunction. To address electrical conduction defects, Zhang et al. [122] developed a multifunctional hydrogel incorporating poly(3,4-ethylenedioxythiophene):poly(styrenesulfonate), α-tocopherol, and PDA. This system synergistically enhances cardiomyocyte regeneration and restores contractile performance through electroactive microenvironment modulation. Addressing surgical invasiveness limitations, Liang et al. [123] proposed a conductive hydrogel that achieves conformal myocardial adhesion through autonomous interfacial interactions. Under the action of Fe^3+^, the team synthesized polypyrrole nanoparticle-embedded hydrogels via copolymerization of pyrrole monomers with water-soluble hyperbranched poly(amino ester), establishing a novel translational approach for myocardial repair. Inspired by the natural properties of swim bladders, Song et al. [124] developed a novel hydrogel characterized by high flexibility, anisotropic alignment, conductivity, and structural similarity to cardiac tissue, designed for post-MI repair.

The past decade has seen remarkable progress in developing therapeutic hydrogels for MI, yielding innovative systems such as injectable formulations and topical patches with enhanced biointegration capabilities. Nevertheless, the complex pathophysiology of post-MI tissue remodeling—encompassing inflammation, fibrosis, and electromechanical dysregulation—poses critical limitations for single-target intervention approaches. Current hydrogel platforms exhibit constrained therapeutic efficacy, primarily focusing on discrete biological processes (e.g., inflammatory regulation or neovascularization) rather than comprehensive pathway modulation. This paradigm underscores the urgent need for next-generation multifunctional hydrogels capable of spatiotemporal regulation of the post-MI microenvironment through smart responsive mechanisms. Concurrent optimization of viscoelastic properties remains crucial to achieve myocardial tissue-mimetic mechanical behavior while maintaining essential injectability.

### Micelles

Micelles represent nanoscale drug delivery systems formed via spontaneous self-assembly of amphiphilic block copolymers in aqueous media [125]. These self-organized supramolecular nanostructures feature a well-defined core–shell morphology, with hydrophobic domains segregating to form a drug-loading core and hydrophilic moieties stabilizing the outer-corona interface. This structural duality provides 3 key therapeutic advantages: enhanced hydrosolubility, scalable fabrication processes, and biocompatible profiles. These characteristics position micellar platforms as versatile drug delivery systems capable of dispersing and transporting hydrophobic therapeutics with limited aqueous solubility.

**Fig. 13.**
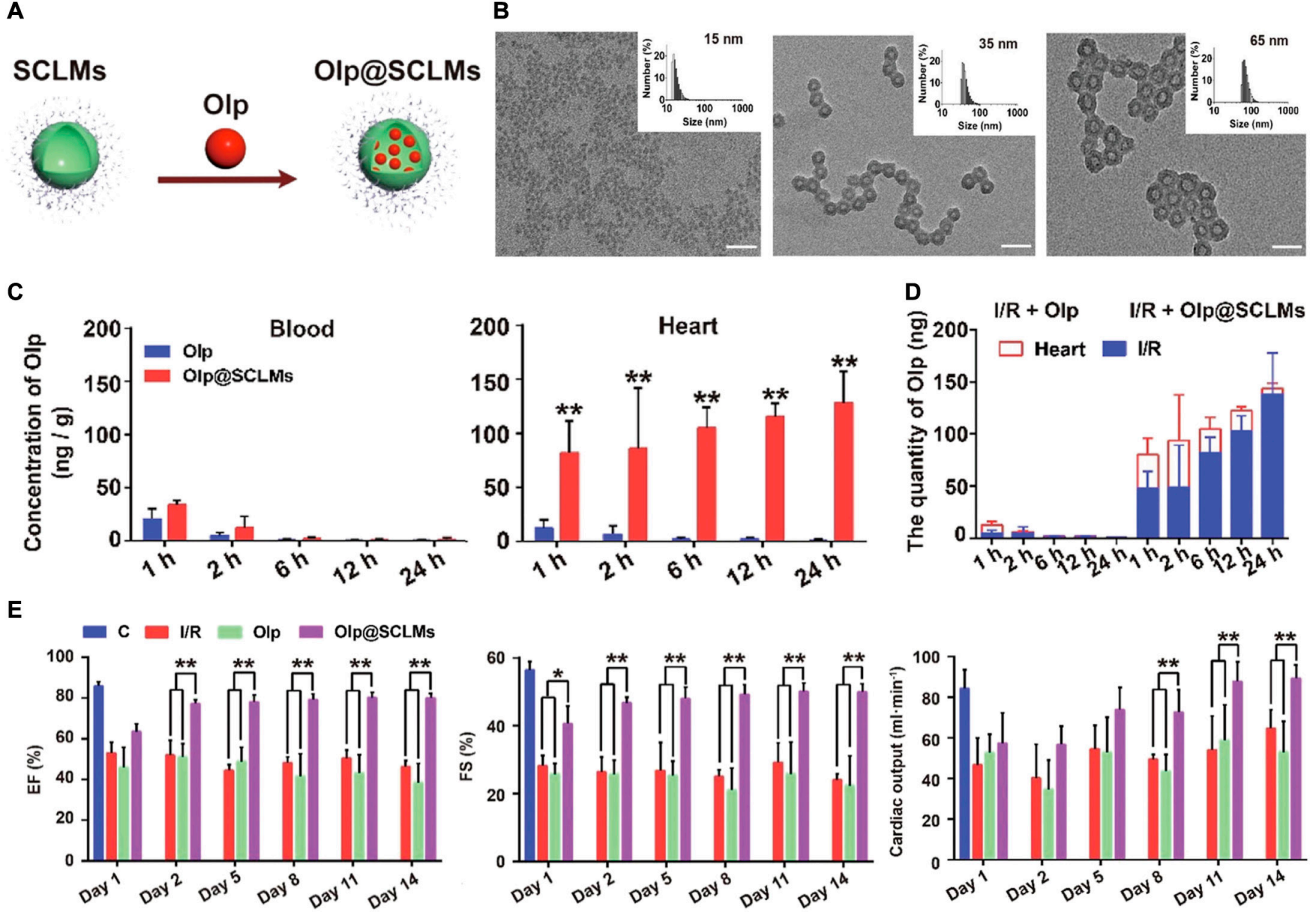
(A) Schematic illustration of Olp@SCLM fabrication. (B) TEM images of SCLMs with different sizes (C) Distribution of Olp in the blood and heart of the MIRI rats determined by high-performance liquid chromatography–mass spectrometry (HPLC-MS) using milrinone as the internal reference. (D) Amount of Olp in the whole heart (open red bar) and ischemia–reperfusion myocardium (solid blue bar) during 1 to 24 h after injection. (E) Main cardiac functions determined by echocardiography. Reproduced with permission [126]. Copyright 2022, American Chemical Society.

Olprinone (Olp), a clinically approved cardiotonic agent, increases intracellular cyclic adenosine monophosphate levels by selectively inhibiting phosphodiesterase III. This mechanism induces dual cardioprotective effects: improved myocardial contractility and peripheral vasodilation. However, its therapeutic efficacy is substantially constrained by inherent hydrophobicity and poor pharmacokinetic properties. To address these constraints, Yang et al. [126] developed an innovative hollow-structured silica-crosslinked micellar nanoplatform to encapsulate Olp for the treatment of MIRI (Fig. 13). Mechanistic investigations revealed that the nanomaterial can effectively target damaged myocardium via the enhanced permeability and retention (EPR) effect as well as endocytosis. This synergistic targeting strategy markedly enhanced Olp’s bioavailability and extended its plasma half-life, ultimately potentiating cardioprotective efficacy.

Capitalizing on the pathologically overexpressed matrix metalloproteinase-9 (MMP-9) within MI microenvironments, Wang et al. [127] engineered a stimuli-responsive peptide with dual-phase transitional properties for spatiotemporally controlled bFGF delivery. This rationally designed amphiphilic peptide is capable of forming micelles in vitro and has MMP-9-specific cleavage sites, which can achieve sustained release of bFGF through structural reconstruction. Furthermore, certain hydrophobic herbal components possessing therapeutic effects on cardiovascular diseases, such as TSA, astragaloside IV, and resveratrol, also employ micelles as drug delivery systems to ameliorate MIRI.

At the same time, to dynamically evaluate antioxidant therapies, Shi et al. [128] developed a theranostic micellar nanoprobe with dual-modal imaging capacity. It was fabricated by modifying the ONOO^−^-responsive NIR fluorescent dye and Cy3 on silica-crosslinked micelles (SCLMs), which facilitates real-time monitoring of the ROS content during coronary artery reperfusion.

While micelles have shown promising therapeutic potential in MIRI treatment, several critical challenges remain to be addressed in their clinical translation. Micelles size (10 to 100 nm) critically modulate biodistribution and target tissue accumulation by exploiting the EPR effect, essential for efficient drug delivery. However, current triblock copolymer-based systems demonstrate constrained structural flexibility due to their characteristic molecular architecture, preventing controlled expansion beyond predetermined size thresholds. Consequently, this limitation impedes micelle size optimization for maximal EPR efficacy. Additionally, effective EPR-mediated targeting requires micelles to have both an appropriate size and a low critical micelle concentration (CMC). This low CMC is essential to maintain structural integrity and stability during circulation, enabling effective drug delivery.

### Lipid-based nanomaterials

Lipid-based nanomaterials—particularly liposomes and lipid nanoparticles (LNPs)—represent key targeted delivery platforms for MIRI therapy. While both systems employ lipid constituents, distinct structural and functional properties dictate their specialized therapeutic applications. Featuring phospholipid bilayer structures, liposomes encapsulate and protect labile therapeutics (e.g., antioxidant enzymes, nucleic acids, and peptides). Their targeting capability to ischemic myocardium is achieved via passive mechanisms (leveraging the EPR effect) or active strategies employing myocardial targeting antibodies or cell-penetrating peptides. Wang et al. [129] demonstrated enhanced efficacy with dual-modified liposomes: IMTP mediates tissue-specific accumulation, while TPP enables mitochondrial delivery. This achieves subcellular targeting of therapeutics (e.g., PUE), mitigating MIRI-associated mitochondrial impairment. Dong et al. [130] advanced liposomal therapy by developing spleen-targeted melatonin liposomes (ST-MT@Lipo2) to treat MIRI through cardiosplenic axis modulation. These liposomes induce polarization of splenic monocytes/macrophages toward anti-inflammatory M2 phenotypes and inhibit the MCP-1/CCR2 axis, reducing inflammatory monocyte migration to the myocardium. Consequently, myocardial inflammation is attenuated and cardiac function is improved at the source.

Conversely, LNPs exhibit complex multi-component architectures incorporating ionizable lipids, cholesterol, helper phospholipids, and PEGylated lipids. These form dense hydrophobic cores that enable efficient encapsulation and delivery of nucleic acid therapeutics (e.g., siRNA and mRNA). This structural configuration confers superior endosomal escape capacity, establishing LNPs as ideal vectors for gene therapy and RNA-based intervention in MIRI pathological cascades. Recent studies confirm that LNPs modified with CMPs efficiently deliver cardioprotective long noncoding RNA Oip5-as1 to the heart [131]. Intravenous administration of these LNPs inhibits the p53 signaling pathway, preserves mitochondrial function, attenuates cardiomyocyte apoptosis, markedly reduces infarct size, and improves cardiac function. In a separate study, Handa et al. [132] employed transcatheter intracoronary injection to deliver mRNA-loaded LNPs. This approach overcomes limitations of intravenous administration (low targeting efficiency) and intramyocardial injection (substantial tissue trauma and limited diffusion), enabling efficient and uniform therapeutic mRNA expression [e.g., vascular endothelial growth factor (VEGF) mRNA] throughout the myocardium.

Despite substantial advances, clinical translation of lipid-based nanomaterials for MIRI faces challenges such as inconsistent targeting efficiency (particularly in systemic delivery), off-target accumulation, and long-term safety risks including immunogenicity. Future research needs to focus on developing intelligent stimuli-responsive carriers, optimizing delivery pathways, and gaining a deeper understanding of nanomaterial–biointerface interactions. While clinical translation demands rigorous standardization, safety validation, and efficacy verification, accelerated technological progress—exemplified by approved liposomal therapeutics—foreshadows viable clinical implementation pathways for MIRI management.

### Polymeric nanomaterials

Polymer nanomaterials represent a class of multifunctional drug delivery platforms engineered from synthetic or natural macromolecules. Tunable physicochemical properties—molecular weight, biodegradability, and surface functionality—enable precise modulation of drug-loading capacity, release kinetics, and targeting specificity. These attributes position polymeric nanomaterials as ideal systems for addressing the pathological cascade in MIRI. The HI@PSeP-IMTP system exemplifies this potential: a PLGA-based nanoprobe functionalized with IMTP and ROS-responsive diselenide bonds [133]. This system achieves precise targeting to MIRI sites while enabling real-time self-monitoring through NIR fluorescence and photoacoustic imaging. Furthermore, it synergistically ameliorates injury by combining diselenide bond-mediated ROS scavenging with hesperadin’s anti-inflammatory action, ultimately improving cardiac function and reducing inflammatory markers. To address hypoxic microenvironments, polymer-based oxygen-releasing nanoparticles (e.g., PCNP/O₂) enable targeted and sustained oxygen delivery via intravenous administration, including prophylactic application prior to MI onset [134]. This noninvasive system selectively accumulates in infarcted myocardium, markedly enhancing cardiomyocyte survival, attenuating fibrosis, and ultimately restoring cardiac function.

The inherent design flexibility of polymeric nanomaterials provides an ideal foundation for integrating advanced drug delivery strategies. For instance, combining polymeric nanomaterials with cell membrane encapsulation imparts nanomaterials with enhanced biomimetic capabilities [68,70,135]. This functionalization prolongs systemic circulation while leveraging membrane protein-mediated targeting and inflammatory tropism to enhance active accumulation in ischemic myocardium. Similarly, polymeric nanomaterials serve as core scaffolds for integrating exosomes, aptamers, or multi-response triggers to develop sophisticated theranostic systems with synergistic functions. Future efforts should prioritize developing multifunctional carriers capable of microenvironment-responsive behavior and deep tissue penetration. Integrating complementary technologies will accelerate development of clinical-grade nanomedicines with scalable production, advancing their translation toward personalized MIRI therapies.

In summary, biomimetic, inorganic, hydrogel, micelle, lipid-based, and polymeric nanomaterials demonstrate distinct advantages for MIRI therapy, although inherent limitations persist (Table 3). Core material properties—particularly targeting efficiency and biocompatibility—critically determine clinical translatability.

**Table 3.** Advantages and disadvantages of various nanomaterials for MIRI therapy

Type		Advantages	Disadvantages
Biomimetic nanomaterials	Monocytes/macrophages	Inherent inflammatory tropism Immune-modulating capacity	Limited drug loading Potential immunogenicity
	Neutrophils	Enhanced inflammatory tropism Cytokine neutralization	Risk of inflammatory amplification Short circulation half-life
	Platelets	Natural thrombus affinity Prolonged circulation	Residual thrombogenicity risk Restricted targeting specificity
	Hybrid membrane	Multifunctional targeting Synergistic biointeractions	Complex fabrication Potential immunogenicity amplification
	Exosome-based	Low immunogenicity Efficient biological barrier penetration	Low drug-loading efficiency Batch-to-batch variability
	Lipoprotein-mimetic	Natural targeting via specific receptors Endogenous lipid transport mimicry	Limited research specifically for MIRI Complex in vivo metabolic pathways
Inorganic nanomaterials	Metallic	Unique physicochemical properties High surface reactivity	Potential long-term toxicity Aggregation tendency
	Nonmetallic	Excellent biocompatibility Tunable porosity for drug loading	Slow/incomplete biodegradation
Hydrogel	/	Excellent biocompatibility Mechanical support for tissue repair	Complex administration Potential swelling-induced volume changes
Micelles	/	Scalable fabrication Size-tunable for EPR effect	Thermodynamic instability Limited drug capacity
Lipid-based nanomaterials	Liposomes	Versatile cargo protection Clinically established	Oxidation susceptibility Poor batch consistency in scale-up
	LNPs	Superior nucleic acid delivery Efficient endosomal escape	Complex formulation Limited long-term safety data
Polymeric nanomaterials	/	Highly tunable properties Stimuli-responsive design	Potential for incomplete degradation Insufficient long-term biosafety data

## Conclusions and Future Perspectives

**Fig. 14.**
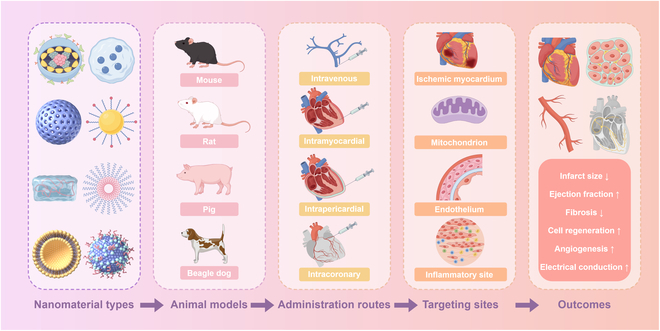
Schematic overview of nanomaterial-based strategies for MIRI therapy. (By Figdraw.)

Current first-line cardioprotective agents—including β-blockers for cardiac load reduction and angiotensin-converting enzyme (ACE) inhibitors suppressing ventricular remodeling—primarily act via systemic neurohormonal regulation. However, suboptimal ischemic site bioavailability (<5% cardiac accumulation) and dose-limiting systemic toxicity (e.g., hypotension and renal impairment) constrain their efficacy. In contrast, nanomaterial-based therapeutics have emerged as a paradigm-shifting strategy for MIRI therapy, demonstrating unparalleled advantages in spatiotemporal drug delivery, multi-mechanism modulation, and bioimaging integration (Table 3). By leveraging engineered size-dependent biodistribution (20- to 50-nm cardiac endothelial penetration), stimuli-responsive payload release, and biomimetic targeting (e.g., platelet membrane-coated systems with >90% cardiac specificity), these platforms have achieved 8- to 12-fold higher infarct zone accumulation compared to free drugs in preclinical models. This strategy achieves 40% to 50% infarct size reduction—significantly surpassing the 15% to 25% reduction observed with β-blockers in comparable models. The targeted approach concurrently enhances cardioprotection while minimizing off-target toxicity. Although direct nanoformulation of β-blockers or ACE inhibitors for MIRI remains underexplored, substantial preclinical evidence confirms that nanoplatforms enhance cardiovascular drug delivery efficiency, offering viable technical solutions. For instance, ROS-responsive hydrogels encapsulating bFGF enable sustained drug release [115], maintaining therapeutic concentrations to overcome the pharmacokinetic limitations of captopril (*t*_1/2_ ≈ 2 h) and similar agents. To systematically integrate nanomaterial strategies for MIRI therapy, we developed a schematic diagram (Fig. 14) that synthesizes 5 key aspects: nanomaterial types, preclinical animal models, administration routes, targeting sites, and therapeutic outcomes—establishing a visual framework for translational cross-species research.

Despite compelling preclinical success, translating nanotherapeutics for MIRI to clinical practice faces urgent multifaceted challenges. First, MIRI’s complex pathophysiology requires interventions targeting interconnected pathways (e.g., inflammation, oxidative stress, calcium overload, and ferroptosis). Current nanoplatforms typically target single pathways, underscoring the need for multi-targeting or combinatorial strategies. Second, critical biosafety issues remain unresolved. Nondegradable components (e.g., Pt-N-C nanozymes with 28-d murine half-lives) raise concerns about chronic toxicity and bioaccumulation. Chronic bioaccumulation may cause fibrosis, organ burden (e.g., hepatic/splenic), or disruption of metal ion homeostasis (e.g., iron). Although biomimetic coatings improve targeting and immune evasion, they carry risks like residual thrombogenicity (platelet membranes) or unintended immunogenicity/inflammation (hybrid/leukocyte membranes). Rigorous assessment of long-term biocompatibility, immunotoxicity, and hypersensitivity potential is essential. Third, scaling complex nanoformulation manufacturing faces reproducibility hurdles. Precise control of critical quality attributes—size distribution, surface charge, ligand density, drug-loading efficiency, and batch consistency—is challenging, particularly for complex designs (e.g., cell membrane fusion, hybrid coatings, and multi-stage targeting). This complexity adversely affects cost-effectiveness and regulatory compliance. Fourth, current research relies predominantly on rodent models and short-term assessments. These models have limited predictive value for human efficacy/safety, especially concerning chronic toxicity, longitudinal immune responses, or performance with MIRI-associated comorbidities (e.g., diabetes and atherosclerosis). Robust validation in physiologically/immunologically relevant large mammalian models remains essential but scarce. Finally, regulatory navigation for these complex novel therapeutics poses unique challenges. Meeting stringent regulatory standards (e.g., U.S. Food and Drug Administration/European Medicines Agency) for clinical trials requires demonstrating consistent quality/safety/efficacy, necessitating substantial investment and innovative characterization methods.

Addressing these translational barriers requires developing intelligent multifunctional nanomaterials that simultaneously target multiple MIRI pathways with integrated real-time diagnostics. Safety-by-design prioritization entails using biodegradable materials, optimizing clearance pathways, minimizing immunogenicity, and incorporating immunomodulatory properties. Concurrently, standardized protocols for long-term toxicity profiling—including chronic exposure, genotoxicity, and organ-specific effects—in large animal models are essential. Rigorous preclinical validation requires MIRI models with comorbidities to evaluate efficacy, biodistribution, pharmacokinetics/pharmacodynamics, and long-term safety. Finally, it is crucial to foster early interdisciplinary collaboration among nanomaterial scientists, cardiologists, immunotoxicologists, regulatory experts, and industry partners to align research with clinical and regulatory requirements.

In conclusion, although nanomaterial-based therapies show undeniable preclinical promise for MIRI, addressing these translational challenges is crucial. Focused research on safety, manufacturability, multi-targeting efficacy, and rigorous large animal validation will unlock the potential of these nanotherapeutics, enabling clinical translation and improving outcomes for MIRI patients.
